# Planning a cluster randomized trial with unequal cluster sizes: practical issues involving continuous outcomes

**DOI:** 10.1186/1471-2288-6-17

**Published:** 2006-04-12

**Authors:** Lydia Guittet, Philippe Ravaud, Bruno Giraudeau

**Affiliations:** 1 Département d'Epidémiologie, Biostatistique et Recherche Clinique, Groupe Hospitalier Bichat-Claude Bernard (AP-HP), Université Xavier Bichat, Paris, France; 2 INSERM U 738, Université Paris 7, Paris, France; 3 INSERM CIC 202; Université François Rabelais de Tours; CHRU de Tours, France; 4 INSERM U 717, Université Paris 7, Paris, France

## Abstract

**Background:**

Cluster randomization design is increasingly used for the evaluation of health-care, screeening or educational interventions. At the planning stage, sample size calculations usually consider an average cluster size without taking into account any potential imbalance in cluster size. However, there may exist high discrepancies in cluster sizes.

**Methods:**

We performed simulations to study the impact of an imbalance in cluster size on power. We determined by simulations to which extent four methods proposed to adapt the sample size calculations to a pre-specified imbalance in cluster size could lead to adequately powered trials.

**Results:**

We showed that an imbalance in cluster size can be of high influence on the power in the case of severe imbalance, particularly if the number of clusters is low and/or the intraclass correlation coefficient is high. In the case of a severe imbalance, our simulations confirmed that the minimum variance weights correction of the variation inflaction factor (VIF) used in the sample size calculations has the best properties.

**Conclusion:**

Publication of cluster sizes is important to assess the real power of the trial which was conducted and to help designing future trials. We derived an adaptation of the VIF from the minimum variance weights correction to be used in case the imbalance can be a priori formulated such as "a proportion (*γ*) of clusters actually recruit a proportion (*τ*) of subjects to be included (*γ *≤ *τ*)".

## Background

A cluster randomized trial involves randomizing social units or clusters of individuals rather than the individuals themselves. This design, which is increasingly being used for evaluating healthcare, screening and educational interventions presents specific constraints that must be considered during planning and analysis [1,2]. Indeed, the responses of individuals within a cluster tend to be more similar than those of individuals of different clusters, and we thus define the clustering effect as 1 + (*m *- 1)*ρ*, where *m *is the average number of subjects per cluster and *ρ *the intraclass correlation coefficient (ICC). This clustering effect is used during the planning of cluster randomized trials as an inflation factor to increase the sample size required by an individual randomization trial. However, such an approach does not take into account variations in cluster size, which might differ greatly. Indeed, as illustrated by Kerry *et al *[3], cluster size may depend on, for example, (i) the potential of recruitment of the cluster (i.e., the number of subjects belonging to each cluster), (ii) the eligible fraction of subjects, which may vary among clusters, or (iii) the ability of physicians to recruit subjects within each cluster. Such an imbalance in cluster size reduces the power of the trial and has to be taken into account in the sample size calculation.

Kerry *et al *[3] assessed the theoretical efficacy of 3 weightings of the inflation factor but in the context of cluster level analysis, so summary statistics are estimated at the cluster level and the unit of analysis remains the cluster. Manatunga *et al *[4], however, assessed a correction on the basis of the assumed distribution of cluster sizes in the context of marginal models, but the authors' simulations covered a range of ICCs larger than those usually observed in cluster randomized trials.

Our aim was therefore to assess these proposed corrections in the framework of cluster randomized trials in which the unit of analysis remains the subject, embedded in the cluster. We first describe the random effects model used to simulate clustered data; then display the simulation design used to evaluate the loss of power due to imbalance in cluster size and the findings. Corrections of the variance inflation factor to allow for cluster size inequality evaluated by simulation and robustness of these corrections to misspecification of the ICC is assessed. practical guidelines for the planning stage of cluster randomized trials are drawn and perspectives for future research.

## Methods and results

### Theoretical background

#### The mixed effects model

Let us supposed a continuous outcome distributed according to the following mixed-effects model:

*Y*_*ijk *_= *θ*_*i *_+ *β*_*ij *_+ *ε*_*ijk*_    (1)

where *Y*_*ijk *_is the observed response for the *k*th subject in the *j*th cluster of the *i*th group, *θ*_*i *_is the overall mean in the *i*th group, *β*_*ij *_is the random effect associated with the cluster effect and *ε*_*ijk *_is the residual effect. The *β*_*ij *_and *ε*_*ijk *_are assumed to be independent and normally distributed as (0; σb2
 MathType@MTEF@5@5@+=feaafiart1ev1aaatCvAUfKttLearuWrP9MDH5MBPbIqV92AaeXatLxBI9gBaebbnrfifHhDYfgasaacH8akY=wiFfYdH8Gipec8Eeeu0xXdbba9frFj0=OqFfea0dXdd9vqai=hGuQ8kuc9pgc9s8qqaq=dirpe0xb9q8qiLsFr0=vr0=vr0dc8meaabaqaciaacaGaaeqabaqabeGadaaakeaaiiGacqWFdpWCdaqhaaWcbaGaemOyaigabaGaeGOmaidaaaaa@30E2@) and (0; σw2
 MathType@MTEF@5@5@+=feaafiart1ev1aaatCvAUfKttLearuWrP9MDH5MBPbIqV92AaeXatLxBI9gBaebbnrfifHhDYfgasaacH8akY=wiFfYdH8Gipec8Eeeu0xXdbba9frFj0=OqFfea0dXdd9vqai=hGuQ8kuc9pgc9s8qqaq=dirpe0xb9q8qiLsFr0=vr0=vr0dc8meaabaqaciaacaGaaeqabaqabeGadaaakeaaiiGacqWFdpWCdaqhaaWcbaGaem4DaChabaGaeGOmaidaaaaa@310C@) respectively.

The ICC quantifies the degree of similarity between the responses of subjects in the same cluster and is defined as the proportion of the total outcome variation between clusters:

ρ=σb2σb2+σw2     (2)
 MathType@MTEF@5@5@+=feaafiart1ev1aaatCvAUfKttLearuWrP9MDH5MBPbIqV92AaeXatLxBI9gBaebbnrfifHhDYfgasaacH8akY=wiFfYdH8Gipec8Eeeu0xXdbba9frFj0=OqFfea0dXdd9vqai=hGuQ8kuc9pgc9s8qqaq=dirpe0xb9q8qiLsFr0=vr0=vr0dc8meaabaqaciaacaGaaeqabaqabeGadaaakeaaiiGacqWFbpGCcqGH9aqpdaWcaaqaaiab=n8aZnaaDaaaleaacqWGIbGyaeaacqaIYaGmaaaakeaacqWFdpWCdaqhaaWcbaGaemOyaigabaGaeGOmaidaaOGaey4kaSIae83Wdm3aa0baaSqaaiabdEha3bqaaiabikdaYaaaaaGccaWLjaGaaCzcamaabmaabaGaeGOmaidacaGLOaGaayzkaaaaaa@40F0@

#### Sample size calculations

Considering *g *clusters of *m *individuals to be randomized in each group, the total number of subject *N *per group is given by [2]:

N=mg=2σ2(t(1−α/2),2(g−1)+t(1−β),2(g−1))2[1+(m−1)ρ]Δ2     (3)
 MathType@MTEF@5@5@+=feaafiart1ev1aaatCvAUfKttLearuWrP9MDH5MBPbIqV92AaeXatLxBI9gBaebbnrfifHhDYfgasaacH8akY=wiFfYdH8Gipec8Eeeu0xXdbba9frFj0=OqFfea0dXdd9vqai=hGuQ8kuc9pgc9s8qqaq=dirpe0xb9q8qiLsFr0=vr0=vr0dc8meaabaqaciaacaGaaeqabaqabeGadaaakeaacqWGobGtcqGH9aqpcqWGTbqBcqWGNbWzcqGH9aqpdaWcaaqaaiabikdaYGGaciab=n8aZnaaCaaaleqabaGaeGOmaidaaOWaaeWaaeaacqWG0baDdaWgaaWcbaWaaeWaaeaacqaIXaqmcqGHsislcqWFXoqycqGGVaWlcqaIYaGmaiaawIcacaGLPaaacqGGSaalcqaIYaGmdaqadaqaaiabdEgaNjabgkHiTiabigdaXaGaayjkaiaawMcaaaqabaGccqGHRaWkcqWG0baDdaWgaaWcbaWaaeWaaeaacqaIXaqmcqGHsislcqWFYoGyaiaawIcacaGLPaaacqGGSaalcqaIYaGmdaqadaqaaiabdEgaNjabgkHiTiabigdaXaGaayjkaiaawMcaaaqabaaakiaawIcacaGLPaaadaahaaWcbeqaaiabikdaYaaakmaadmaabaGaeGymaeJaey4kaSYaaeWaaeaacqWGTbqBcqGHsislcqaIXaqmaiaawIcacaGLPaaacqWFbpGCaiaawUfacaGLDbaaaeaacqqHuoardaahaaWcbeqaaiabikdaYaaaaaGccaWLjaGaaCzcamaabmaabaGaeG4mamdacaGLOaGaayzkaaaaaa@66FE@

where Δ is the absolute mean difference between groups (i.e., Δ = |*θ*_0 _- *θ*_1_|), *σ*^2 ^is the total variance defined as (σb2
 MathType@MTEF@5@5@+=feaafiart1ev1aaatCvAUfKttLearuWrP9MDH5MBPbIqV92AaeXatLxBI9gBaebbnrfifHhDYfgasaacH8akY=wiFfYdH8Gipec8Eeeu0xXdbba9frFj0=OqFfea0dXdd9vqai=hGuQ8kuc9pgc9s8qqaq=dirpe0xb9q8qiLsFr0=vr0=vr0dc8meaabaqaciaacaGaaeqabaqabeGadaaakeaaiiGacqWFdpWCdaqhaaWcbaGaemOyaigabaGaeGOmaidaaaaa@30E2@ + σw2
 MathType@MTEF@5@5@+=feaafiart1ev1aaatCvAUfKttLearuWrP9MDH5MBPbIqV92AaeXatLxBI9gBaebbnrfifHhDYfgasaacH8akY=wiFfYdH8Gipec8Eeeu0xXdbba9frFj0=OqFfea0dXdd9vqai=hGuQ8kuc9pgc9s8qqaq=dirpe0xb9q8qiLsFr0=vr0=vr0dc8meaabaqaciaacaGaaeqabaqabeGadaaakeaaiiGacqWFdpWCdaqhaaWcbaGaem4DaChabaGaeGOmaidaaaaa@310C@) and *t*_(1 - *α*/2),2(*g *- 1) _and *t*_(1 - *β*),2(*g *- 1) _is the 100 × (1 - *α*/2) and 100 × (1 - *β*) percentiles of the Student *t*-distribution with 2(*g *- 1) degrees of freedom. Considering the effect size, defined as the relative difference between groups (i.e., *ES *= |*θ*_0 _- *θ*_1_|/*σ *= Δ/*σ*), expression (3) can be re-written as:

N=2(t(1−α/2),2(g−1)+t(1−β),2(g−1))2[1+(m−1)ρ]ES2     (4)
 MathType@MTEF@5@5@+=feaafiart1ev1aaatCvAUfKttLearuWrP9MDH5MBPbIqV92AaeXatLxBI9gBaebbnrfifHhDYfgasaacH8akY=wiFfYdH8Gipec8Eeeu0xXdbba9frFj0=OqFfea0dXdd9vqai=hGuQ8kuc9pgc9s8qqaq=dirpe0xb9q8qiLsFr0=vr0=vr0dc8meaabaqaciaacaGaaeqabaqabeGadaaakeaacqWGobGtcqGH9aqpdaWcaaqaaiabikdaYmaabmaabaGaemiDaq3aaSbaaSqaamaabmaabaGaeGymaeJaeyOeI0ccciGae8xSdeMaei4la8IaeGOmaidacaGLOaGaayzkaaGaeiilaWIaeGOmaiZaaeWaaeaacqWGNbWzcqGHsislcqaIXaqmaiaawIcacaGLPaaaaeqaaOGaey4kaSIaemiDaq3aaSbaaSqaamaabmaabaGaeGymaeJaeyOeI0Iae8NSdigacaGLOaGaayzkaaGaeiilaWIaeGOmaiZaaeWaaeaacqWGNbWzcqGHsislcqaIXaqmaiaawIcacaGLPaaaaeqaaaGccaGLOaGaayzkaaWaaWbaaSqabeaacqaIYaGmaaGcdaWadaqaaiabigdaXiabgUcaRmaabmaabaGaemyBa0MaeyOeI0IaeGymaedacaGLOaGaayzkaaGae8xWdihacaGLBbGaayzxaaaabaGaemyrauKaem4uam1aaWbaaSqabeaacqaIYaGmaaaaaOGaaCzcaiaaxMaadaqadaqaaiabisda0aGaayjkaiaawMcaaaaa@6135@

### Impact of cluster size inequality

#### Simulation study

Monte Carlo simulations were used to assess the impact of imbalance in cluster size on both power and type I error. A 2 × 4 × 4 factorial plan was used, considering 2 effect sizes (0.25, 0.50) to be detected with fixed numbers of clusters (5, 10, 20, 40) and 4 *a priori *postulated values of the ICC (0.005, 0.02, 0.05, 0.10). The ICC values were chosen according to previously published estimates [5-15], and the number of clusters is in agreement with that from a recent review of cluster randomized trials in primary care settings in which the median number of randomized clusters was estimated at 34 [13]. The *α *and *β *values were fixed at 0.05 and 0.20, respectively, in any case.

Once the sample size was calculated, correlated data were simulated, according to model (1). From a practical point of view, data were generated as the sum of a fixed effect (*θ*_0 _or *θ*_1 _if the control or experimental group, respectively) and realizations of the 2 random variables *β*_*ij *_and *ε*_*ijk*_. For convenience and without loss of generality we set *θ*_0 _equal to 0 and (σb2
 MathType@MTEF@5@5@+=feaafiart1ev1aaatCvAUfKttLearuWrP9MDH5MBPbIqV92AaeXatLxBI9gBaebbnrfifHhDYfgasaacH8akY=wiFfYdH8Gipec8Eeeu0xXdbba9frFj0=OqFfea0dXdd9vqai=hGuQ8kuc9pgc9s8qqaq=dirpe0xb9q8qiLsFr0=vr0=vr0dc8meaabaqaciaacaGaaeqabaqabeGadaaakeaaiiGacqWFdpWCdaqhaaWcbaGaemOyaigabaGaeGOmaidaaaaa@30E2@ + σw2
 MathType@MTEF@5@5@+=feaafiart1ev1aaatCvAUfKttLearuWrP9MDH5MBPbIqV92AaeXatLxBI9gBaebbnrfifHhDYfgasaacH8akY=wiFfYdH8Gipec8Eeeu0xXdbba9frFj0=OqFfea0dXdd9vqai=hGuQ8kuc9pgc9s8qqaq=dirpe0xb9q8qiLsFr0=vr0=vr0dc8meaabaqaciaacaGaaeqabaqabeGadaaakeaaiiGacqWFdpWCdaqhaaWcbaGaem4DaChabaGaeGOmaidaaaaa@310C@) equal to 1. These constraints then allow for defining *θ*_1 _as the effect size ES, σb2
 MathType@MTEF@5@5@+=feaafiart1ev1aaatCvAUfKttLearuWrP9MDH5MBPbIqV92AaeXatLxBI9gBaebbnrfifHhDYfgasaacH8akY=wiFfYdH8Gipec8Eeeu0xXdbba9frFj0=OqFfea0dXdd9vqai=hGuQ8kuc9pgc9s8qqaq=dirpe0xb9q8qiLsFr0=vr0=vr0dc8meaabaqaciaacaGaaeqabaqabeGadaaakeaaiiGacqWFdpWCdaqhaaWcbaGaemOyaigabaGaeGOmaidaaaaa@30E2@ as *ρ *and σw2
 MathType@MTEF@5@5@+=feaafiart1ev1aaatCvAUfKttLearuWrP9MDH5MBPbIqV92AaeXatLxBI9gBaebbnrfifHhDYfgasaacH8akY=wiFfYdH8Gipec8Eeeu0xXdbba9frFj0=OqFfea0dXdd9vqai=hGuQ8kuc9pgc9s8qqaq=dirpe0xb9q8qiLsFr0=vr0=vr0dc8meaabaqaciaacaGaaeqabaqabeGadaaakeaaiiGacqWFdpWCdaqhaaWcbaGaem4DaChabaGaeGOmaidaaaaa@310C@ as (1 - *ρ*).

#### Cluster size

For any combination of *ES*, *g *and *ρ*, we simulated randomized trials with, on the one hand, constant cluster size and, on the other, imbalance in cluster size. In the absence of cluster sizes publications, three types of imbalance were considered:

1. A moderate imbalance:

For each group, each of the *N *subjects had an equiprobability of being in any of the *g *clusters randomized in this group. From a practical point of view, for any of the *N *subjects, we randomly selected with equiprobability the cluster to which it belongs, before adding the appropriate realizations of random variables *β*_*ij *_and *ε*_*ijk*_.

2. A "Pareto" imbalance

Following the economic Pareto's principle, we considered the situation in which 80% of the subjects actually belong to only 20% of the clusters. From a practical point of view, we thus defined 2 strata within each group: the strata of large clusters (e.g., 20% of the *g *clusters) and the strata of small clusters. Eighty percent of the *N *subjects were in the large cluster strata, while the 20% remaining were in the small cluster strata. Then, within each stratum, subjects were randomly assigned with equiprobability to one of the clusters.

3. A Poisson imbalance

Cluster sizes were finally defined according to a Poisson distribution, which has already been used in such a context [16,17]. We thus considered a Poisson distribution with parameter *m *defined as *N*/*g *and defined the cluster size of any cluster before generating the associated observations.

In this latter situation, and contrary to the 2 previous ones, the total number of patients per group varies and is equal to *N *only on average. Moreover, in the 3 types of cluster size inequality, the actual number of clusters per group could be smaller than *g*, because clusters could be empty.

For any combination of ES, *g *and ICC, and for any situation (balance or any type of imbalance in cluster size), 5000 replications of data were simulated by use of SAS 8.1 software.

#### Analysis

Data analysis involved no stratification on cluster size. We used the MIXED procedure in SAS [18,19] to assess restricted maximum likelihood (REML) estimates of variance components. The Wald test statistic was then used to test the significance of the intervention effect with the Student t-distribution, with g_0_+g_1_-2 degrees of freedom as the reference distribution, where g_0 _and g_1 _are the actual numbers of nonempty clusters in the control and intervention groups, respectively.

The empirical type I error and power were calculated as the proportion of significant trials (defined as a p value smaller than the nominal *α *level) when *θ*_1 _equals 0 and *ES*, respectively.

## Results

Results are expressed as absolute bias and mean square error on the one hand, and empirical' type I error and power on the other. Table 1 displays the results associated with an *a priori *postulated effect size of 0.25, while Table 2 displays the results associated with a 0.50 effect size. In 7 situations, data sets could not be generated for the following combinations ES/ICC/g: 0.25/0.020/5, 0.25/0.050/5, 0.25/0.050/10, 0.25/0.100/5, 0.25/0.100/10, 0.25/0.100/20 and 0.50/0.100/5. Indeed, when the number of clusters is small and/or the ICC high, even an infinite cluster size may not allow for achieving 80% power [20].

**Table 1:** Bias, mean square error, empirical type I error and power in cluster randomized trials according to several types of imbalance in cluster size – Effect size = 0.25

**Simulation parameters**^1^			**Type of imbalance**	**Bias**	**Mean Square Error**	**Empirical type I error**^2^	**Empirical power**^2^
					
**Intraclass correlation coefficient (*ρ*)**	**Number of clusters in each arm (*g*)**	**Total number of subjects in each arm (*N*)**					
**0.005**	**5**	**485**	**None**	-0.0020	0.0062	0.0328	0.7756
			**Moderate**	-0.0015	0.0062	0.0300	0.7800
			**Poisson**	0.0003	0.0061	0.0368	0.7814
			**Pareto**	0.0002	0.0100	0.0664	0.6432
							
**0.005**	**10**	**326**	**None**	0.0005	0.0070	0.0326	0.7868
			**Moderate**	0.0009	0.0073	0.0402	0.7838
			**Poisson**	-0.0010	0.0070	0.0356	0.7884
			**Pareto**	0.0043	0.0100	0.0566	0.6968
							
**0.005**	**20**	**282**	**None**	-0.0010	0.0072	0.0320	0.7942
			**Moderate**	0.0014	0.0075	0.0398	0.7878
			**Poisson**	-0.0010	0.0076	0.0408	0.7802
			**Pareto**	0.0000	0.0090	0.0486	0.7258
							
**0.005**	**40**	**265**	**None**	0.0006	0.0078	0.0444	0.7848
			**Moderate**	0.0011	0.0082	0.0458	0.7936
			**Poisson**	-0.0017	0.0082	0.0484	0.7772
			**Pareto**	0.0000	0.0086	0.0466	0.7572
							
**0.020**	**10**	**629**	**None**	-0.0017	0.0070	0.0448	0.8012
			**Moderate**	0.0017	0.0073	0.0544	0.7974
			**Poisson**	0.0009	0.0074	0.0510	0.7992
			**Pareto**	-0.0022	0.0118	0.0904	0.6236
							
**0.020**	**20**	**353**	**None**	-0.0004	0.0073	0.0452	0.8000
			**Moderate**	0.0006	0.0074	0.0408	0.7980
			**Poisson**	-0.0007	0.0075	0.0458	0.7968
			**Pareto**	0.0009	0.0115	0.0660	0.6546
							
**0.020**	**40**	**290**	**None**	0.0017	0.0080	0.0518	0.7932
			**Moderate**	0.0001	0.0077	0.0466	0.7944
			**Poisson**	-0.0003	0.0077	0.0466	0.7912
			**Pareto**	0.0003	0.0101	0.0556	0.7008
							
**0.050**	**20**	**743**	**None**	-0.0007	0.0075	0.0436	0.7916
			**Moderate**	-0.0018	0.0077	0.0540	0.8026
			**Poisson**	-0.0003	0.0078	0.0536	0.7950
			**Pareto**	-0.0022	0.0115	0.0562	0.6256
							
**0.050**	**40**	**361**	**None**	-0.0012	0.0080	0.0528	0.7944
			**Moderate**	0.0031	0.0080	0.0510	0.7926
			**Poisson**	-0.0001	0.0080	0.0502	0.7904
			**Pareto**	-0.0023	0.0121	0.0604	0.6242
							
**0.100**	**40**	**652**	**None**	0.0021	0.0076	0.0504	0.7966
			**Moderate**	0.0013	0.0078	0.0458	0.8118
			**Poisson**	-0.0022	0.0078	0.0506	0.7946
			**Pareto**	-0.0031	0.0121	0.0546	0.6006

^1^N is the number of subjects per intervention arm, calculated under the assumption of constant cluster size

^2 ^The nominal values for type I and type II error rates were fixed at 0.05 and 0.20, respectively.

**Table 2:** Bias, mean square error, empirical type I error and power in cluster randomized trials according to several types of imbalance in cluster size – Effect size = 0.50

**Simulation parameters**^1^			**Type of imbalance**	**Bias**	**Mean Square Error**	**Empirical type I error**^2^	**Empirical power**^2^
					
**Intraclass correlation coefficient (*ρ*)**	**Number of clusters in each arm (*g*)**	**Total number of subjects in each arm (*N*)**	**Type of imbalance**				
**0.005**	**5**	**89**	**None**	0.0025	0.0238	0.0190	0.7648
			**Moderate**	0.0010	0.0243	0.0204	0.7622
			**Poisson**	0.0014	0.0243	0.0214	0.7596
			**Pareto**	-0.0064	0.0393	0.0256	0.6250
							
**0.005**	**10**	**73**	**None**	-0.0011	0.0288	0.0328	0.7660
			**Moderate**	-0.0015	0.0290	0.0322	0.7718
			**Poisson**	-0.0007	0.0298	0.0318	0.7662
			**Pareto**	-0.0005	0.0344	0.0352	0.7090
							
**0.005**	**20**	**67**	**None**	-0.0011	0.0303	0.0384	0.7764
			**Moderate**	-0.0038	0.0296	0.0318	0.7700
			**Poisson**	-0.0012	0.0301	0.0382	0.7664
			**Pareto**	-0.0004	0.0323	0.0334	0.7322
							
**0.005**	**40**	**65**	**None**	0.0005	0.0310	0.0446	0.7986
			**Moderate**	0.0021	0.0322	0.0478	0.7896
			**Poisson**	0.0028	0.0305	0.0396	0.7860
			**Pareto**	-0.0007	0.0320	0.0382	0.7518
							
**0.020**	**5**	**119**	**None**	0.0025	0.0238	0.0190	0.7648
			**Moderate**	-0.0011	0.0248	0.0310	0.7856
			**Poisson**	-0.0021	0.0250	0.0306	0.7786
			**Pareto**	0.0009	0.0413	0.0674	0.6262
							
**0.020**	**10**	**81**	**None**	-0.0031	0.0273	0.0320	0.7798
			**Moderate**	0.0006	0.0282	0.0364	0.7772
			**Poisson**	-0.0003	0.0288	0.0378	0.7778
			**Pareto**	0.0078	0.0394	0.0550	0.6910
							
**0.020**	**20**	**70**	**None**	0.0026	0.0312	0.0476	0.7838
			**Moderate**	0.0026	0.0312	0.0476	0.7838
			**Poisson**	0.0032	0.0306	0.0436	0.7894
			**Pareto**	0.0003	0.0362	0.0460	0.7056
							
**0.020**	**40**	**66**	**None**	-0.0026	0.0314	0.0498	0.7878
			**Moderate**	0.0026	0.0312	0.0476	0.7838
			**Poisson**	0.0049	0.0326	0.0476	0.7828
			**Pareto**	-0.0007	0.0337	0.0422	0.7328
							
**0.050**	**5**	**423**	**None**	0.0015	0.0246	0.0482	0.7980
			**Moderate**	-0.0001	0.0240	0.0460	0.8004
			**Poisson**	-0.0005	0.0237	0.0478	0.7988
			**Pareto**	0.0026	0.0337	0.0768	0.6808
							
**0.050**	**10**	**103**	**None**	-0.0006	0.0280	0.0426	0.7964
			**Moderate**	0.0012	0.0286	0.0446	0.7952
			**Poisson**	-0.0017	0.0293	0.0440	0.7754
			**Pareto**	-0.0022	0.0466	0.0770	0.6342
							
**0.050**	**20**	**76**	**None**	0.0027	0.0298	0.0436	0.8020
			**Moderate**	-0.0018	0.0308	0.0452	0.7784
			**Poisson**	-0.0016	0.0323	0.0526	0.7672
			**Pareto**	-0.0056	0.0396	0.0528	0.6620
							
**0.050**	**40**	**67**	**None**	-0.0019	0.0313	0.0468	0.7880
			**Moderate**	-0.0012	0.0294	0.0516	0.7740
			**Poisson**	0.0000	0.0335	0.0504	0.7712
			**Pareto**	0.0022	0.0376	0.0516	0.7026
							
**0.100**	**10**	**213**	**None**	0.0006	0.0263	0.0426	0.8056
			**Moderate**	0.0015	0.0289	0.0530	0.7940
			**Poisson**	-0.0007	0.0287	0.0538	0.8004
			**Pareto**	-0.0027	0.0438	0.0730	0.6394
							
**0.100**	**20**	**89**	**None**	-0.0029	0.0303	0.0470	0.7888
			**Moderate**	-0.0020	0.0324	0.0530	0.7760
			**Poisson**	-0.0004	0.0316	0.0488	0.7744
			**Pareto**	0.0064	0.0492	0.0674	0.6276
							
**0.100**	**40**	**70**	**None**	0.0038	0.0331	0.0510	0.7890
			**Moderate**	0.0031	0.0337	0.0506	0.7738
			**Poisson**	0.0020	0.0332	0.0456	0.7658
			**Pareto**	0.0019	0.0433	0.0536	0.6641

^1^N is the number of subjects per intervention arm, calculated under the assumption of constant cluster size

^2 ^The nominal values for type I and type II error rates were fixed at 0.05 and 0.20, respectively.

No significant bias was induced by inequality in cluster size (since the relative bias was no more than about 1.5%, in absolute value), while the mean square error was barely increased in cases of severe imbalance (Pareto imbalance).

When the number of clusters is small, type I errors were estimated at a lower level than the nominal one, even with no imbalance in cluster sizes. A symmetrical result was also observed for power, which was estimated at a lower level than the nominal one. This result was of greater magnitude for small ICCs and for greater effect size, which corresponded to situations in which the total number of subjects to be included is reduced. Otherwise, although moderate and Poisson imbalances were of no influence, a Pareto's imbalance was associated with an increase in both type I and type II errors. As an example, if one is willing to detect a 0.25 effect size and plan a randomized trial with 10 clusters per arm with an *a priori *postulated ICC of 0.02, a Pareto imbalance leads to type I and type II errors of 9% and 38%, respectively, and nominal values fixed at 5% and 20%. This result is of greater magnitude for large ICCs and a small number of clusters.

Thus, while moderate imbalances (based on an equiprobability hypothesis) and Poisson's imbalances can be neglected at the planning stage, a more severe imbalance (such as the Pareto's imbalance) should be taken into account, thus leading to an adjustment in sample size calculations.

### Sample size adjustment for unbalanced trials

#### Adjusted variance inflation factors

The (1 + (*m *- 1)*ρ*) factor in expressions (3) and (4) defines the variance inflation factor (VIF) that takes into account the correlation induced by the cluster randomization. This VIF supposes a constant cluster size (*m*) or is based on the average cluster size in case of imbalance. Kerry *et al *[3] and Manatunga *et al *[4] proposed to adjust the VIF in cases of an imbalance in cluster size. Thus, we propose 4 corrections. The first 3 are based on weights derived from the *a priori *postulated distribution of cluster sizes among the g clusters (i.e., the different values of *m*_*j*_, where *m*_*j *_is the size of the *j*^th ^cluster), and the fourth is based on the expected mean and variance of this latter distribution.

1. Equal weights (denoted *w*_1_)[3]:

VIFw1=m¯g∑j=1g1mj(1−ρ)+m¯ρ
 MathType@MTEF@5@5@+=feaafiart1ev1aaatCvAUfKttLearuWrP9MDH5MBPbIqV92AaeXatLxBI9gBaebbnrfifHhDYfgasaacH8akY=wiFfYdH8Gipec8Eeeu0xXdbba9frFj0=OqFfea0dXdd9vqai=hGuQ8kuc9pgc9s8qqaq=dirpe0xb9q8qiLsFr0=vr0=vr0dc8meaabaqaciaacaGaaeqabaqabeGadaaakeaacqWGwbGvcqWGjbqscqWGgbGrdaWgaaWcbaGaem4DaC3aaSbaaWqaaiabigdaXaqabaaaleqaaOGaeyypa0ZaaSaaaeaacuWGTbqBgaqeaaqaaiabdEgaNbaadaaeWbqaamaalaaabaGaeGymaedabaGaemyBa02aaSbaaSqaaiabdQgaQbqabaaaaOWaaeWaaeaacqaIXaqmcqGHsisliiGacqWFbpGCaiaawIcacaGLPaaacqGHRaWkcuWGTbqBgaqeaiab=f8aYbWcbaGaemOAaOMaeyypa0JaeGymaedabaGaem4zaCganiabggHiLdaaaa@4AF5@ where m¯=1g∑j=1gmj
 MathType@MTEF@5@5@+=feaafiart1ev1aaatCvAUfKttLearuWrP9MDH5MBPbIqV92AaeXatLxBI9gBaebbnrfifHhDYfgasaacH8akY=wiFfYdH8Gipec8Eeeu0xXdbba9frFj0=OqFfea0dXdd9vqai=hGuQ8kuc9pgc9s8qqaq=dirpe0xb9q8qiLsFr0=vr0=vr0dc8meaabaqaciaacaGaaeqabaqabeGadaaakeaacuWGTbqBgaqeaiabg2da9maalaaabaGaeGymaedabaGaem4zaCgaamaaqahabaGaemyBa02aaSbaaSqaaiabdQgaQbqabaaabaGaemOAaOMaeyypa0JaeGymaedabaGaem4zaCganiabggHiLdaaaa@3B51@

2. Cluster size weights (denoted *w*_2_)[3]:

VIFw2=1+(mA¯−1)ρ
 MathType@MTEF@5@5@+=feaafiart1ev1aaatCvAUfKttLearuWrP9MDH5MBPbIqV92AaeXatLxBI9gBaebbnrfifHhDYfgasaacH8akY=wiFfYdH8Gipec8Eeeu0xXdbba9frFj0=OqFfea0dXdd9vqai=hGuQ8kuc9pgc9s8qqaq=dirpe0xb9q8qiLsFr0=vr0=vr0dc8meaabaqaciaacaGaaeqabaqabeGadaaakeaacqWGwbGvcqWGjbqscqWGgbGrdaWgaaWcbaGaem4DaC3aaSbaaWqaaiabikdaYaqabaaaleqaaOGaeyypa0JaeGymaeJaey4kaSIaeiikaGYaa0aaaeaacqWGTbqBdaWgaaWcbaGaemyqaeeabeaaaaGccqGHsislcqaIXaqmcqGGPaqkiiGacqWFbpGCaaa@3DCB@ where mA¯=∑j=1gmj2∑j=1gmj
 MathType@MTEF@5@5@+=feaafiart1ev1aaatCvAUfKttLearuWrP9MDH5MBPbIqV92AaeXatLxBI9gBaebbnrfifHhDYfgasaacH8akY=wiFfYdH8Gipec8Eeeu0xXdbba9frFj0=OqFfea0dXdd9vqai=hGuQ8kuc9pgc9s8qqaq=dirpe0xb9q8qiLsFr0=vr0=vr0dc8meaabaqaciaacaGaaeqabaqabeGadaaakeaadaqdaaqaaiabd2gaTnaaBaaaleaacqWGbbqqaeqaaaaakiabg2da9maalaaabaWaaabCaeaacqWGTbqBdaqhaaWcbaGaemOAaOgabaGaeGOmaidaaaqaaiabdQgaQjabg2da9iabigdaXaqaaiabdEgaNbqdcqGHris5aaGcbaWaaabCaeaacqWGTbqBdaWgaaWcbaGaemOAaOgabeaaaeaacqWGQbGAcqGH9aqpcqaIXaqmaeaacqWGNbWza0GaeyyeIuoaaaaaaa@450E@

3. Minimum variance weights (denoted *w*_3_) [3]:

VIFw3=m¯g∑j=1gmj1+(mj−1)ρ
 MathType@MTEF@5@5@+=feaafiart1ev1aaatCvAUfKttLearuWrP9MDH5MBPbIqV92AaeXatLxBI9gBaebbnrfifHhDYfgasaacH8akY=wiFfYdH8Gipec8Eeeu0xXdbba9frFj0=OqFfea0dXdd9vqai=hGuQ8kuc9pgc9s8qqaq=dirpe0xb9q8qiLsFr0=vr0=vr0dc8meaabaqaciaacaGaaeqabaqabeGadaaakeaacqWGwbGvcqWGjbqscqWGgbGrdaWgaaWcbaGaem4DaC3aaSbaaWqaaiabiodaZaqabaaaleqaaOGaeyypa0ZaaSaaaeaacuWGTbqBgaqeaiabdEgaNbqaamaaqahabaWaaSaaaeaacqWGTbqBdaWgaaWcbaGaemOAaOgabeaaaOqaaiabigdaXiabgUcaRmaabmaabaGaemyBa02aaSbaaSqaaiabdQgaQbqabaGccqGHsislcqaIXaqmaiaawIcacaGLPaaaiiGacqWFbpGCaaaaleaacqWGQbGAcqGH9aqpcqaIXaqmaeaacqWGNbWza0GaeyyeIuoaaaaaaa@4AB9@

4. Distribution-based correction (denoted d) [4]:

VIFd=1+(E(m)2+var⁡(m)E(m)−1)ρ
 MathType@MTEF@5@5@+=feaafiart1ev1aaatCvAUfKttLearuWrP9MDH5MBPbIqV92AaeXatLxBI9gBaebbnrfifHhDYfgasaacH8akY=wiFfYdH8Gipec8Eeeu0xXdbba9frFj0=OqFfea0dXdd9vqai=hGuQ8kuc9pgc9s8qqaq=dirpe0xb9q8qiLsFr0=vr0=vr0dc8meaabaqaciaacaGaaeqabaqabeGadaaakeaacqWGwbGvcqWGjbqscqWGgbGrdaWgaaWcbaGaemizaqgabeaakiabg2da9iabigdaXiabgUcaRmaabmaabaWaaSaaaeaacqWGfbqrdaqadaqaaiabd2gaTbGaayjkaiaawMcaamaaCaaaleqabaGaeGOmaidaaOGaey4kaSIagiODayNaeiyyaeMaeiOCai3aaeWaaeaacqWGTbqBaiaawIcacaGLPaaaaeaacqWGfbqrdaqadaqaaiabd2gaTbGaayjkaiaawMcaaaaacqGHsislcqaIXaqmaiaawIcacaGLPaaaiiGacqWFbpGCaaa@4ACE@

where E(*m*) and var(*m*) are the expected mean and the variance of the cluster size.

We considered these 4 adjustments when a Pareto's imbalance is *a priori *supposed to be observed. Since moderate imbalances have been shown to be of no influence, we assumed a constant cluster size within each stratum associated with the Pareto's imbalance. The adjusted VIF then becomes (Appendix A):

VIFw1=3.25+(m¯w1−3.25)ρ     (5)
 MathType@MTEF@5@5@+=feaafiart1ev1aaatCvAUfKttLearuWrP9MDH5MBPbIqV92AaeXatLxBI9gBaebbnrfifHhDYfgasaacH8akY=wiFfYdH8Gipec8Eeeu0xXdbba9frFj0=OqFfea0dXdd9vqai=hGuQ8kuc9pgc9s8qqaq=dirpe0xb9q8qiLsFr0=vr0=vr0dc8meaabaqaciaacaGaaeqabaqabeGadaaakeaacqWGwbGvcqWGjbqscqWGgbGrdaWgaaWcbaGaem4DaC3aaSbaaWqaaiabigdaXaqabaaaleqaaOGaeyypa0JaeG4mamJaeiOla4IaeGOmaiJaeGynauJaey4kaSYaaeWaaeaacuWGTbqBgaqeamaaBaaaleaacqWG3bWDdaWgaaadbaGaeGymaedabeaaaSqabaGccqGHsislcqaIZaWmcqGGUaGlcqaIYaGmcqaI1aqnaiaawIcacaGLPaaaiiGacqWFbpGCcaWLjaGaaCzcamaabmaabaGaeGynaudacaGLOaGaayzkaaaaaa@48A4@

with m¯w1=6.5(1−ρ)T2gES2−2ρT2
 MathType@MTEF@5@5@+=feaafiart1ev1aaatCvAUfKttLearuWrP9MDH5MBPbIqV92AaeXatLxBI9gBaebbnrfifHhDYfgasaacH8akY=wiFfYdH8Gipec8Eeeu0xXdbba9frFj0=OqFfea0dXdd9vqai=hGuQ8kuc9pgc9s8qqaq=dirpe0xb9q8qiLsFr0=vr0=vr0dc8meaabaqaciaacaGaaeqabaqabeGadaaakeaacuWGTbqBgaqeamaaBaaaleaacqWG3bWDdaWgaaadbaGaeGymaedabeaaaSqabaGccqGH9aqpdaWcaaqaaiabiAda2iabc6caUiabiwda1maabmaabaGaeGymaeJaeyOeI0ccciGae8xWdihacaGLOaGaayzkaaGaemivaq1aaWbaaSqabeaacqaIYaGmaaaakeaacqWGNbWzcqWGfbqrcqWGtbWudaahaaWcbeqaaiabikdaYaaakiabgkHiTiabikdaYiab=f8aYjabdsfaunaaCaaaleqabaGaeGOmaidaaaaaaaa@471B@ and *T *= *t*_(1 - *α*/2),2(*g *- 1) _+ *t*_(1 - *β*),2(*g *- 1)_

VIFw2=1+(3.25m¯w2−1)ρ     (6)
 MathType@MTEF@5@5@+=feaafiart1ev1aaatCvAUfKttLearuWrP9MDH5MBPbIqV92AaeXatLxBI9gBaebbnrfifHhDYfgasaacH8akY=wiFfYdH8Gipec8Eeeu0xXdbba9frFj0=OqFfea0dXdd9vqai=hGuQ8kuc9pgc9s8qqaq=dirpe0xb9q8qiLsFr0=vr0=vr0dc8meaabaqaciaacaGaaeqabaqabeGadaaakeaacqWGwbGvcqWGjbqscqWGgbGrdaWgaaWcbaGaem4DaC3aaSbaaWqaaiabikdaYaqabaaaleqaaOGaeyypa0JaeGymaeJaey4kaSYaaeWaaeaacqaIZaWmcqGGUaGlcqaIYaGmcqaI1aqncuWGTbqBgaqeamaaBaaaleaacqWG3bWDdaWgaaadbaGaeGOmaidabeaaaSqabaGccqGHsislcqaIXaqmaiaawIcacaGLPaaaiiGacqWFbpGCcaWLjaGaaCzcamaabmaabaGaeGOnaydacaGLOaGaayzkaaaaaa@46C8@

with m¯w2=2(1−ρ)T2gES2−6.5ρT2
 MathType@MTEF@5@5@+=feaafiart1ev1aaatCvAUfKttLearuWrP9MDH5MBPbIqV92AaeXatLxBI9gBaebbnrfifHhDYfgasaacH8akY=wiFfYdH8Gipec8Eeeu0xXdbba9frFj0=OqFfea0dXdd9vqai=hGuQ8kuc9pgc9s8qqaq=dirpe0xb9q8qiLsFr0=vr0=vr0dc8meaabaqaciaacaGaaeqabaqabeGadaaakeaacuWGTbqBgaqeamaaBaaaleaacqWG3bWDdaWgaaadbaGaeGOmaidabeaaaSqabaGccqGH9aqpdaWcaaqaaiabikdaYmaabmaabaGaeGymaeJaeyOeI0ccciGae8xWdihacaGLOaGaayzkaaGaemivaq1aaWbaaSqabeaacqaIYaGmaaaakeaacqWGNbWzcqWGfbqrcqWGtbWudaahaaWcbeqaaiabikdaYaaakiabgkHiTiabiAda2iabc6caUiabiwda1iab=f8aYjabdsfaunaaCaaaleqabaGaeGOmaidaaaaaaaa@471D@

VIFw3=(1+(4m¯w3−1)ρ)(1+(0.25m¯w3−1)ρ)1+(m¯w3−1)ρ     (7)
 MathType@MTEF@5@5@+=feaafiart1ev1aaatCvAUfKttLearuWrP9MDH5MBPbIqV92AaeXatLxBI9gBaebbnrfifHhDYfgasaacH8akY=wiFfYdH8Gipec8Eeeu0xXdbba9frFj0=OqFfea0dXdd9vqai=hGuQ8kuc9pgc9s8qqaq=dirpe0xb9q8qiLsFr0=vr0=vr0dc8meaabaqaciaacaGaaeqabaqabeGadaaakeaacqWGwbGvcqWGjbqscqWGgbGrdaWgaaWcbaGaem4DaC3aaSbaaWqaaiabiodaZaqabaaaleqaaOGaeyypa0ZaaSaaaeaadaqadaqaaiabigdaXiabgUcaRmaabmaabaGaeGinaqJafmyBa0MbaebadaWgaaWcbaGaem4DaC3aaSbaaWqaaiabiodaZaqabaaaleqaaOGaeyOeI0IaeGymaedacaGLOaGaayzkaaacciGae8xWdihacaGLOaGaayzkaaWaaeWaaeaacqaIXaqmcqGHRaWkdaqadaqaaiabicdaWiabc6caUiabikdaYiabiwda1iqbd2gaTzaaraWaaSbaaSqaaiabdEha3naaBaaameaacqaIZaWmaeqaaaWcbeaakiabgkHiTiabigdaXaGaayjkaiaawMcaaiab=f8aYbGaayjkaiaawMcaaaqaaiabigdaXiabgUcaRmaabmaabaGafmyBa0MbaebadaWgaaWcbaGaem4DaC3aaSbaaWqaaiabiodaZaqabaaaleqaaOGaeyOeI0IaeGymaedacaGLOaGaayzkaaGae8xWdihaaiaaxMaacaWLjaWaaeWaaeaacqaI3aWnaiaawIcacaGLPaaaaaa@616E@

with m¯w3
 MathType@MTEF@5@5@+=feaafiart1ev1aaatCvAUfKttLearuWrP9MDH5MBPbIqV92AaeXatLxBI9gBaebbnrfifHhDYfgasaacH8akY=wiFfYdH8Gipec8Eeeu0xXdbba9frFj0=OqFfea0dXdd9vqai=hGuQ8kuc9pgc9s8qqaq=dirpe0xb9q8qiLsFr0=vr0=vr0dc8meaabaqaciaacaGaaeqabaqabeGadaaakeaacuWGTbqBgaqeamaaBaaaleaacqWG3bWDdaWgaaadbaGaeG4mamdabeaaaSqabaaaaa@30F6@ being the positive solution of the following equation:

m¯w32ρ[gES2−2ρT2]+m¯w3(1−ρ)[gES2−8.5ρT2]−2(1−ρ)2T2=0
 MathType@MTEF@5@5@+=feaafiart1ev1aaatCvAUfKttLearuWrP9MDH5MBPbIqV92AaeXatLxBI9gBaebbnrfifHhDYfgasaacH8akY=wiFfYdH8Gipec8Eeeu0xXdbba9frFj0=OqFfea0dXdd9vqai=hGuQ8kuc9pgc9s8qqaq=dirpe0xb9q8qiLsFr0=vr0=vr0dc8meaabaqaciaacaGaaeqabaqabeGadaaakeaacuWGTbqBgaqeamaaDaaaleaacqWG3bWDdaWgaaadbaGaeG4mamdabeaaaSqaaiabikdaYaaaiiGakiab=f8aYnaadmaabaGaem4zaCMaemyrauKaem4uam1aaWbaaSqabeaacqaIYaGmaaGccqGHsislcqaIYaGmcqWFbpGCcqWGubavdaahaaWcbeqaaiabikdaYaaaaOGaay5waiaaw2faaiabgUcaRiqbd2gaTzaaraWaaSbaaSqaaiabdEha3naaBaaameaacqaIZaWmaeqaaaWcbeaakmaabmaabaGaeGymaeJaeyOeI0Iae8xWdihacaGLOaGaayzkaaWaamWaaeaacqWGNbWzcqWGfbqrcqWGtbWudaahaaWcbeqaaiabikdaYaaakiabgkHiTiabiIda4iabc6caUiabiwda1iab=f8aYjabdsfaunaaCaaaleqabaGaeGOmaidaaaGccaGLBbGaayzxaaGaeyOeI0IaeGOmaiZaaeWaaeaacqaIXaqmcqGHsislcqWFbpGCaiaawIcacaGLPaaadaahaaWcbeqaaiabikdaYaaakiabdsfaunaaCaaaleqabaGaeGOmaidaaOGaeyypa0JaeGimaadaaa@65C0@

VIFd=1+[3.25m¯d−1]ρ     (8)
 MathType@MTEF@5@5@+=feaafiart1ev1aaatCvAUfKttLearuWrP9MDH5MBPbIqV92AaeXatLxBI9gBaebbnrfifHhDYfgasaacH8akY=wiFfYdH8Gipec8Eeeu0xXdbba9frFj0=OqFfea0dXdd9vqai=hGuQ8kuc9pgc9s8qqaq=dirpe0xb9q8qiLsFr0=vr0=vr0dc8meaabaqaciaacaGaaeqabaqabeGadaaakeaacqWGwbGvcqWGjbqscqWGgbGrdaWgaaWcbaGaemizaqgabeaakiabg2da9iabigdaXiabgUcaRmaadmaabaGaeG4mamJaeiOla4IaeGOmaiJaeGynauJafmyBa0MbaebadaWgaaWcbaGaemizaqgabeaakiabgkHiTiabigdaXaGaay5waiaaw2faaGGaciab=f8aYjaaxMaacaWLjaWaaeWaaeaacqaI4aaoaiaawIcacaGLPaaaaaa@4495@

with m¯d=2(1−ρ)T2gES2−6.5ρT2
 MathType@MTEF@5@5@+=feaafiart1ev1aaatCvAUfKttLearuWrP9MDH5MBPbIqV92AaeXatLxBI9gBaebbnrfifHhDYfgasaacH8akY=wiFfYdH8Gipec8Eeeu0xXdbba9frFj0=OqFfea0dXdd9vqai=hGuQ8kuc9pgc9s8qqaq=dirpe0xb9q8qiLsFr0=vr0=vr0dc8meaabaqaciaacaGaaeqabaqabeGadaaakeaacuWGTbqBgaqeamaaBaaaleaacqWGKbazaeqaaOGaeyypa0ZaaSaaaeaacqaIYaGmdaqadaqaaiabigdaXiabgkHiTGGaciab=f8aYbGaayjkaiaawMcaaiabdsfaunaaCaaaleqabaGaeGOmaidaaaGcbaGaem4zaCMaemyrauKaem4uam1aaWbaaSqabeaacqaIYaGmaaGccqGHsislcqaI2aGncqGGUaGlcqaI1aqncqWFbpGCcqWGubavdaahaaWcbeqaaiabikdaYaaaaaaaaa@45CD@

The distribution-based and cluster size weights correction are equivalent [21]. We therefore no longer consider the distribution-based correction and focus on the 3 weighted corrections proposed by Kerry *et al *[3].

#### Simulation study

Monte Carlo simulations were performed to determine to what extent the proposed corrections could lead to adequately powered trials. We thus calculated the sample size needed assuming a Pareto repartition, using each of the adjusted VIFs. For each situation, we then simulated cluster randomized trials with a Pareto imbalance to estimate empirical type I error and power. The same approach as that explained in the preceeding was used.

#### Results

Results are displayed in Tables 3 and 4 for effect sizes of 0.25 and 0.50, respectively. For the cluster size weights correction, several situations existed in which the sample size calculations showed that 80% power could not be reached, thus preventing the generation of associated data sets. If sample size calculations were possible, this correction led to sample sizes barely greater than the sample size obtained with the minimal variance weights correction and empirical type I error and power near the nominal value. This result is consistent for the different values of ES, *ρ *and g in Tables 3 and 4, except for the combination 0.25/0.02/20. Actually, for fixed values of ES, couples of values for (g, *ρ*) lead to null values of the denominator of *m*_*w*2_. If ES is fixed at 0.25, the couple (20, 0.0233) is one of these. For *ρ *just under this critical value (0.020 in our case), m_w2 _begins to diverge, and when *ρ *is greater, m_w2 _can no longer be calculated. Equal weights correction led to a much greater sample size than minimum variance weights, particularly when the ICC is small, and the empirical power obtained was therefore much higher than its nominal value: it may even reach 99% if the nominal value were fixed at 80%. The minimum variance weights correction required the smallest increase in sample size and resulted in the smallest difference between empirical and nominal power. Empirical type I errors were also near the nominal 5% level, except when both the number of clusters and the ICC are small.

### Robustness of sample size adjustment for unbalanced trials with misspecification of the ICC

#### Method

We assessed the robustness of the different sample size adjustments for Pareto-like unbalanced trials with misspecification of the ICC. We considered an effect size of 0.25, *a priori *postulated ICCs of 0.005 and 0.020 and the combinations of number of clusters and cluster sizes previously used (see sample sizes in Table 3). Then, for each weighting method, (i.e., for each total number of subjects of each arm N_w1_, N_w2_, N_w3_) we plotted the expected power calculated for a pre-specified ICC as a function of the real ICC (which will be *a posteriori *assessed). This power was calculated by use of the variance inflation factor VIF_w3 _derived from minimum variance weights, because it allows for calculating an expected power that does not differ from the empirical one by more than 3.8% in the situations explored in Table 3 (data not shown). For reference, we also plotted the expected power (calculated with the usual VIF) as a function of the real ICC in cases of no imbalance in cluster size.

**Table 3:** Required sample size and empirical Type I error and power when using corrected variance inflation factors with an *a priori *hypothesized Pareto imbalance in cluster size – Effect size = 0.25

		**No correction**			**Equal weights**			**Cluster size weights**^1^			**Minimum variance weights**		
**Intraclass correlation coefficient (*ρ*)**	**Number of clusters in each arm (*g*)**	**Sample size**	**Empirical probabilities**		**Sample size**	**Empirical probabilities**		**Sample size**	**Empirical probabilities**		**Sample size**	**Empirical probabilities**	
									
			**Type I error**	**Power**		**Type I error**	**Power**		**Type I error**	**Power**		**Type I error**	**Power**

**0.005**	**5**	485	0.0664	0.6432	1569	0.1028	0.8606	-	-	-	1037	0.0948	0.7992
	**10**	326	0.0566	0.6968	1057	0.0784	0.9386	515	0.0704	0.8106	464	0.0704	0.7806
	**20**	282	0.0486	0.7258	917	0.0624	0.9770	336	0.0450	0.7934	331	0.0458	0.7850
	**40**	265	0.0466	0.7572	861	0.0512	0.9918	287	0.0424	0.7842	286	0.0474	0.7706
													
**0.02**	**10**	629	0.0904	0.6236	2043	0.0638	0.8196	-	-	-	1731	0.0624	0.7968
	20	353	0.0660	0.6546	1147	0.0614	0.8924	1852	0.0576	0.9432	677	0.0752	0.7976
	**40**	290	0.0556	0.7008	942	0.0582	0.9486	435	0.0558	0.8092	401	0.0514	0.7960
													
**0.05**	**20**	743	0.0562	0.6256	2414	0.0564	0.8140	-	-	-	2165	0.0480	0.7976
	**40**	361	0.0604	0.6242	1173	0.0500	0.8598	-	-	-	770	0.0550	0.8048
													
**0.10**	40	652	0.0546	0.6006	2116	0.0542	0.8090	-	-	-	1881	0.0500	0.8036

Sample size calculations were performed considering type I and type II error rates fixed at 0.05 and 0.20, respectively.

^1 ^In some cases, 80% power was not reachable

**Table 4:** Required sample size and empirical Type I error and power when using corrected variance inflation factors with an *a priori *hypothesized Pareto imbalance in cluster size – Effect size = 0.50

		**No correction**			**Equal weights**			**Cluster size weights**^1^			**Minimum variance weights**		
**Intraclass correlation coefficient (*ρ*)**	**Number of clusters in each arm (*g*)**	**Sample size**	**Empirical probabilities**		**Sample size**	**Empirical probabilities**		**Sample size**	**Empirical probabilities**		**Sample size**	**Empirical probabilities**	
									
			**Type I error**	**Power**		**Type I error**	**Power**		**Type I error**	**Power**		**Type I error**	**Power**

**0.005**	**5**	89	0.0256	0.6250	288	0.0536	0.9330	111	0.0270	0.7156	108	0.0324	0.6906
	**10**	73	0.0352	0.7090	236	0.0528	0.9768	79	0.0306	0.7370	79	0.0306	0.7370
	**20**	67	0.0334	0.7322	218	0.0470	0.9912	70	0.0390	0.7524	70	0.0390	0.7524
	**40**	65	0.0382	0.7518	210	0.0394	0.9970	66	0.0400	0.7558	66	0.0400	0.7558
													
**0.02**	**5**	119	0.0674	0.6262	387	0.1072	0.8642	-	-	-	256	0.0954	0.7946
	**10**	81	0.0550	0.6910	261	0.0900	0.9346	127	0.0654	0.7990	115	0.0672	0.7856
	**20**	70	0.0460	0.7056	226	0.0684	0.9752	83	0.0492	0.7680	82	0.0482	0.7680
	**40**	66	0.0422	0.7328	212	0.0534	0.9908	71	0.0390	0.7540	71	0.0390	0.7540
													
**0.05**	**5**	423	0.0768	0.6808	1375	0.0578	0.8130	-	-	-	1311	0.0556	0.7962
	**10**	103	0.0770	0.6342	335	0.0824	0.8600	-	-	-	230	0.0920	0.7952
	**20**	76	0.0528	0.6620	245	0.0652	0.9284	136	0.0706	0.8252	115	0.0628	0.7872
	**40**	67	0.0516	0.7026	217	0.0590	0.9712	83	0.0578	0.7694	81	0.0488	0.7772
													
**0.15**	**10**	213	0.0730	0.6394	691	0.0548	0.8042	-	-	-	631	0.0572	0.8002
	**20**	89	0.0674	0.6276	290	0.0644	0.8646	-	-	-	193	0.0638	0.7888
	**40**	70	0.0536	0.6641	225	0.0564	0.9316	122	0.0506	0.8208	104	0.0578	0.7838

^1^In some cases, 80% power was not reachable

## Results

**Figure 1:**
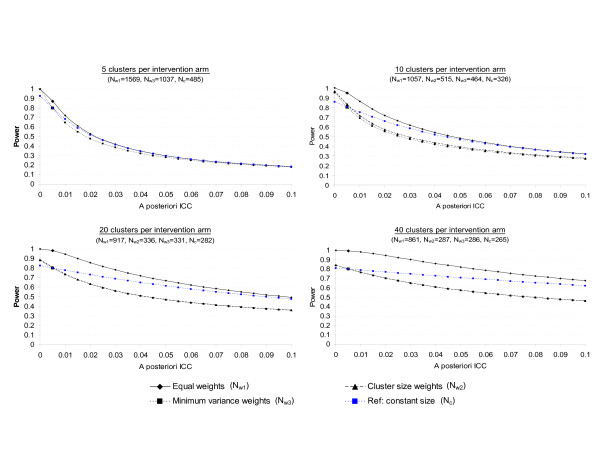
Real power of cluster randomized trials according to the discrepancy between the *a priori *postulated and *a posteriori *estimated intraclass correlation coefficients (ICCs). The ICC is *a priori *postulated at 0.005 and sample sizes (N) and associated powers were calculated: 1°) assuming Pareto repartition of cluster sizes and using 3 corrections of the variance inflation factor (equal weights, cluster size weights and minimum variance weights), 2°) assuming constant cluster size (reference).

**Figure 2:**
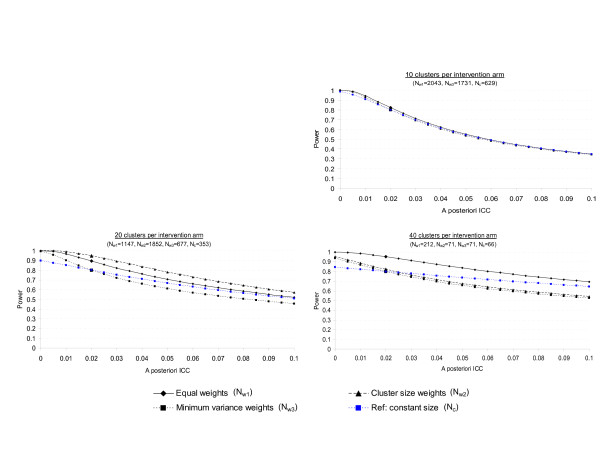
Real power of cluster randomized trials according to the discrepancy between the *a priori *postulated and *a posteriori *estimated intraclass correlation coefficients (ICCs). The ICC is *a priori *postulated at 0.020 and sample sizes (N) and associated powers were calculated: 1°) assuming Pareto repartition of cluster sizes and using 3 corrections of the variance inflation factor (equal weights, cluster size weights and minimum variance weights), 2°) assuming constant cluster size (reference).

Results are displayed in Figures 1 and 2 for an effect size of 0.25 and *a priori *postulated ICC values of 0.005 and 0.020, respectively. As expected [20], in any situation, the power decreases as the ICC increases, and this result is all the more important when the number of clusters is low. In the planning situations explored, minimum variance weights and cluster size weights curves are very close, except when 20 clusters per intervention arm are randomized and the ICC is *a priori *fixed at 0.020, but this latter situation is extreme, as discussed previously. Otherwise, the power associated with equal weights remains greater than that associated with minimum variance weights in any situation. However, this finding probably just reflects that the use of this weighting system leads to higher required sample sizes than the use of a minimum variance weights system (cf Tables 3 and 4) and therefore higher power. In any case, imbalance in cluster size is associated with a higher sensitivity to the a priori-specified ICC than constant cluster size. For example, let us consider the case of 20 clusters per intervention arm: if the ICC is *a priori *postulated at 0.005, but in reality equals 0.015, the power associated with constant cluster size decreases from 0.80 to 0.75 only, whereas the power associated with Pareto repartition decreases from 0.80 to 0.68 (with the minimum variance weighting system). However, all weighting systems show great sensitivity to the actual value of the ICC. Consider the former example (ES = 0.25, g = 20 and Pareto repartition, increase in ICC from 0.005 to 0.015), the power associated with equal weights will decrease from 0.98 to 0.90, and the power associated with cluster size weights from 0.80 to 0.68. Thus, if little prior knowledge is available concerning the value of the ICC, the sensitivity analysis involving several values of ICC is of major importance, particularly when imbalance in cluster size is expected.

### Practical implications

#### General considerations

Cluster size inequality may induce a loss of power and must be taken into account at the planning stage by using the minimum variance weights correction. From a practical point of view, 2 situations must be distinguished. First, when entire clusters are randomized such as in cluster-cluster trials [22]. the cluster size distribution is *a priori *known and cluster size inequalities are therefore easy to be taken into account at the planning stage. Second, if physicians have to recruit patients to each cluster according to selection criteria, cluster size distribution cannot *a priori *be known. In this latter situation, a sensitivity analysis must be performed considering several hypotheses on cluster size distribution for an optimal sample size determination.

#### Adaptation of the VIF for a Pareto like imbalance

Let us assume that the cluster size inequality corresponds to a Pareto-like distribution, say that in each arm a proportion (*γ*) of clusters actually recruit the proportion (*τ*) of patients to be recruited (which implies *γ *≤ *τ*). If *γ *and *τ *are fixed at 20% and 80%, respectively, we have the Pareto imbalance defined previously; if *γ *and *τ *are equal, the cluster size imbalance is absent or moderate (and can then be neglected). The sensitivity analysis then consists of varying the parameters (*γ*) and (*τ*), thus allowing for imbalance increases with the absolute difference between the 2 values. The inflation factor calculated with the minimum variance weights correction will be the following (Appendix B):

VIF=[1+(1−τ1−γm−1)ρ][1+(τγm−1)ρ]τ[1+(1−τ1−γm−1)ρ]+(1−τ)[1+(τγm−1)ρ]     (9)
 MathType@MTEF@5@5@+=feaafiart1ev1aaatCvAUfKttLearuWrP9MDH5MBPbIqV92AaeXatLxBI9gBaebbnrfifHhDYfgasaacH8akY=wiFfYdH8Gipec8Eeeu0xXdbba9frFj0=OqFfea0dXdd9vqai=hGuQ8kuc9pgc9s8qqaq=dirpe0xb9q8qiLsFr0=vr0=vr0dc8meaabaqaciaacaGaaeqabaqabeGadaaakeaacqWGwbGvcqWGjbqscqWGgbGrcqGH9aqpdaWcaaqaamaadmaabaGaeGymaeJaey4kaSYaaeWaaeaadaWcaaqaaiabigdaXiabgkHiTGGaciab=r8a0bqaaiabigdaXiabgkHiTiab=n7aNbaacqWGTbqBcqGHsislcqaIXaqmaiaawIcacaGLPaaacqWFbpGCaiaawUfacaGLDbaadaWadaqaaiabigdaXiabgUcaRmaabmaabaWaaSaaaeaacqWFepaDaeaacqWFZoWzaaGaemyBa0MaeyOeI0IaeGymaedacaGLOaGaayzkaaGae8xWdihacaGLBbGaayzxaaaabaGae8hXdq3aamWaaeaacqaIXaqmcqGHRaWkdaqadaqaamaalaaabaGaeGymaeJaeyOeI0Iae8hXdqhabaGaeGymaeJaeyOeI0Iae83SdCgaaiabd2gaTjabgkHiTiabigdaXaGaayjkaiaawMcaaiab=f8aYbGaay5waiaaw2faaiabgUcaRmaabmaabaGaeGymaeJaeyOeI0Iae8hXdqhacaGLOaGaayzkaaWaamWaaeaacqaIXaqmcqGHRaWkdaqadaqaamaalaaabaGae8hXdqhabaGae83SdCgaaiabd2gaTjabgkHiTiabigdaXaGaayjkaiaawMcaaiab=f8aYbGaay5waiaaw2faaaaacaWLjaGaaCzcamaabmaabaGaeGyoaKdacaGLOaGaayzkaaaaaa@7B24@

To illustrate the discrepancy between nominal and real power if an imbalance of the form "*γ *clusters actually recruit *τ *patients" is not taken into account, we performed the following calculations. We used formula (4) (i.e., assuming a constant cluster size) to derive the number of subjects needed. Then, using expression (9), we calculated the expected power with such a sample size, with a proportion of *γ *clusters actually recruiting a proportion *τ *of the patients to be included.

**Figure 3:**
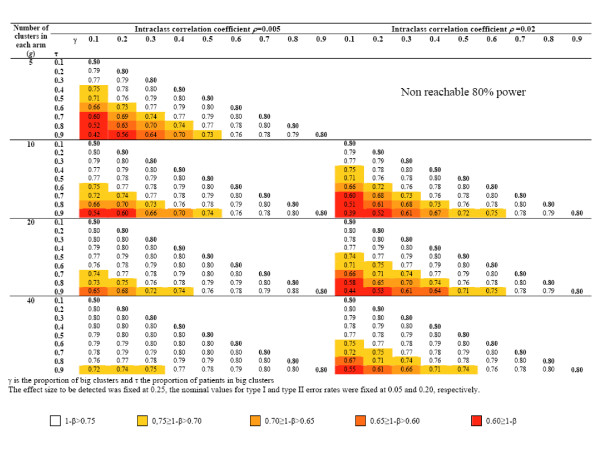
Power of cluster randomized trials if an imbalance in cluster size is not taken into account when planning. The imbalance is *a priori *hypothesized to be "a proportion of *γ *clusters will actually recruit a proportion *τ *of the subjects to be included" (*γ *≤ *τ*) – The intraclass correlation coefficient is fixed at 0.005 and 0.02.

**Figure 4:**
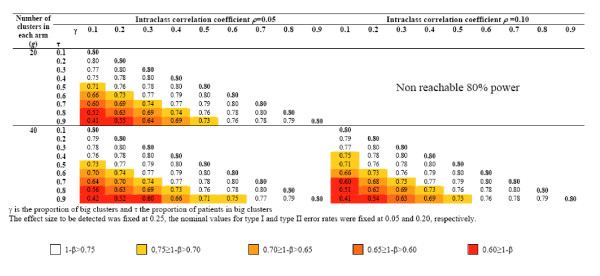
Power of cluster randomized trials if an imbalance in cluster size is not taken into account when planning. The imbalance is *a priori *hypothesized to be "a proportion of *γ *clusters will actually recruit a proportion *τ *of the subjects to be included" (*γ *≤ *τ*) – The intraclass correlation coefficient is fixed at 0.05 and 0.10.

Figures 3 and 4 display the results for several combinations of ES/ICC/g and *γ*/*τ *under the assumption of no empty cluster. The upper part of Figures 3 and 4 is empty, since an 80% power cannot be reach for the associated combinations of ICC and g. Moreover, *γ *is smaller than or equal to *τ*, which explains why any upper part of matrices associated with an ICC/g combination is empty. As expected, the bigger the cluster size inequality, the more important the discrepancy between nominal and real power. For example, let us consider a trial aimed at detecting a 0.25 effect size in which 10 clusters are to be randomized in each arm. Assuming an ICC of 0.005 and a balance in cluster size, this study would require 326 subjects to be recruited in each arm to reach 80% power. If 10% of the clusters recruit 50% of the subjects, the power barely declines, to 77%; if a major imbalance such as 90% of the patients are to be recruited by 10% of the clusters, the power would fall to 54%. The latter phenomenon is all the more acute with a low number of clusters; critical situations in which a substantial loss in power may be expected are displayed in Figures 3 and 4. Red levels approximately follow diagonals representing constant *τ*-*γ *differences. It can be shown (appendix C) that the gini coefficient, a quantitative measure of site accrual inequality [23], comes down to the absolute difference between *τ *and *γ *when a proportion *γ *of clusters actually recruit a proportion *τ *of patients to be recruited. Our results show that varying this summary measure of imbalance is enough for performing a sensitivity analysis and that there is no need to specify both *τ *and *γ*.

Assigning a value of 1 to *τ *creates a situation in which a proportion (1-*γ*) of clusters is empty. In this situation achieving the required sample size supposes to increase the average cluster size of the *γ*g clusters by a factor 1/*γ*. However one has to be aware that such a strategy will indeed allow achieving the pre-specified sample size, but it will not allow to reach the nominal power. Indeed it is known that for a fixed total number of subjects, the higher the number of clusters, the higher the power [1] which means that reducing the number of clusters will translate in a loss in power even if the pre-specified sample size is achieved. Therefore, in case it is anticipated that empty clusters may occur, sensitivity analyses have to be conducted using formula (4) on the basis of the hypothesized number of active clusters g' = *γ*g.

## Discussion

A moderate inequality in cluster sizes has little effect on power and can thus be neglected at the planning stage. However, a major imbalance in cluster sizes, like the "Pareto" imbalance, (i.e. 80% of the subjects belong to only 20% of the clusters) is associated with a loss in power, and the phenomenon is all the more important when the number of clusters is low and/or the ICC is high. In these situations, the minimum variance weights correction has good properties and allows for achieving the nominal power. This result, obtained in the extreme situation of a Pareto imbalance, suggests that this correction can be used to derive sample size or power in any situation where, in each group, cluster sizes can be separated in two strata, the small cluster stratum and the big cluster stratum. The higher sensitivity of severely unbalanced trials to the *a priori*-postulated value of the ICC compared to that of balanced trials emphasized the necessity of a sensitivity analysis on this parameter. We derived an adaptation of the VIF, which should be used when the imbalance is *a priori *hypothesized to be "a proportion of *γ *clusters will actually recruit a proportion *τ *of the subjects to be included".

A limit to this approach remains the degree of imbalance being usually difficult to foresee at the planning stage, except when, for instance, families or practices are randomized and clusters as a whole are included in the trial. In these latter situations, one may *a priori *know precisely the cluster size repartition and therefore use the minimum variance weights correction as initially specified by Kerry et al [3]. However, if, within each cluster, the physician has to recruit patients to be included in the trial, cluster size distribution may then be difficult to hypothesize. It is all the more difficult since cluster sizes are usually not reported in published clustered randomized trials. We therefore proposed to consider that cluster sizes distribution can be divided in each arm in two strata: a stratum of small clusters, and another of large clusters. This hypothesis may be debatable. However, since a moderate inequality of cluster size is of minor effect, it seems a rather useful and simple way to consider the risk of cluster size inequality at the planning stage, particularly since no precise data on cluster size inequality are available. Another limitation is that our work focused on normally distributed continuous outcomes. More work is needed to extend our results to non-normal distributions, especially with binary variables. Finally, we restricted our work to cases of no differential recruitment between arms, thus considering that imbalance is the same in the two arms. Such a hypothesis may be questionable in cluster randomized trials: since inclusion is posterior to randomization, this may indeed induce differential recruitment and imbalance in patient characteristics, which may lead to questioning the results of the study [24].

## Conclusion

In conclusion, our study demonstrates that severely imbalanced trials with continuous outcomes may be highly underpowered. If such imbalance in cluster size can be anticipated at the design stage, minimum variance weights correction should be used to inflate the required sample size. *A priori *estimation of the expectable imbalance would be facilitated if more details on cluster sizes were given in published cluster randomized trials, as was recently advised in the extension of the CONSORT statement for cluster randomized trials [25]. Moreover, such publication of cluster sizes would be of particular interest to assess the real power of the trial conducted.

## Competing interests

The author(s) declare that they have no competing interests.

## Authors' contributions

This study was designed by LG, BG and PR. LG performed the statistical analysis and drafted the article, which was then revised by BG and PR.

## Appendix A: corrected variance inflation factor (VIF) for an *a priori *postulated Pareto imbalance

Four corrections have been proposed for adjusting sample size in cases of imbalance in cluster size. Considering the specific situation of a Pareto imbalance, the general form of these corrections can be simplified.

Characteristics of the Pareto imbalance

**Table 5:** 

	Number of clusters by intervention arm	Number of patients belonging to the clusters	Mean cluster size
Small clusters	0.8*g*	0.2m¯ MathType@MTEF@5@5@+=feaafiart1ev1aaatCvAUfKttLearuWrP9MDH5MBPbIqV92AaeXatLxBI9gBaebbnrfifHhDYfgasaacH8akY=wiFfYdH8Gipec8Eeeu0xXdbba9frFj0=OqFfea0dXdd9vqai=hGuQ8kuc9pgc9s8qqaq=dirpe0xb9q8qiLsFr0=vr0=vr0dc8meaabaqaciaacaGaaeqabaqabeGadaaakeaacuWGTbqBgaqeaaaa@2E27@*g*	(0.2m¯ MathType@MTEF@5@5@+=feaafiart1ev1aaatCvAUfKttLearuWrP9MDH5MBPbIqV92AaeXatLxBI9gBaebbnrfifHhDYfgasaacH8akY=wiFfYdH8Gipec8Eeeu0xXdbba9frFj0=OqFfea0dXdd9vqai=hGuQ8kuc9pgc9s8qqaq=dirpe0xb9q8qiLsFr0=vr0=vr0dc8meaabaqaciaacaGaaeqabaqabeGadaaakeaacuWGTbqBgaqeaaaa@2E27@*g*)/(0.8*g*) = 0.25m¯ MathType@MTEF@5@5@+=feaafiart1ev1aaatCvAUfKttLearuWrP9MDH5MBPbIqV92AaeXatLxBI9gBaebbnrfifHhDYfgasaacH8akY=wiFfYdH8Gipec8Eeeu0xXdbba9frFj0=OqFfea0dXdd9vqai=hGuQ8kuc9pgc9s8qqaq=dirpe0xb9q8qiLsFr0=vr0=vr0dc8meaabaqaciaacaGaaeqabaqabeGadaaakeaacuWGTbqBgaqeaaaa@2E27@
Big clusters	0.2*g*	0.8m¯ MathType@MTEF@5@5@+=feaafiart1ev1aaatCvAUfKttLearuWrP9MDH5MBPbIqV92AaeXatLxBI9gBaebbnrfifHhDYfgasaacH8akY=wiFfYdH8Gipec8Eeeu0xXdbba9frFj0=OqFfea0dXdd9vqai=hGuQ8kuc9pgc9s8qqaq=dirpe0xb9q8qiLsFr0=vr0=vr0dc8meaabaqaciaacaGaaeqabaqabeGadaaakeaacuWGTbqBgaqeaaaa@2E27@*g*	(0.8m¯ MathType@MTEF@5@5@+=feaafiart1ev1aaatCvAUfKttLearuWrP9MDH5MBPbIqV92AaeXatLxBI9gBaebbnrfifHhDYfgasaacH8akY=wiFfYdH8Gipec8Eeeu0xXdbba9frFj0=OqFfea0dXdd9vqai=hGuQ8kuc9pgc9s8qqaq=dirpe0xb9q8qiLsFr0=vr0=vr0dc8meaabaqaciaacaGaaeqabaqabeGadaaakeaacuWGTbqBgaqeaaaa@2E27@*g*)/(0.2*g*) = 4m¯ MathType@MTEF@5@5@+=feaafiart1ev1aaatCvAUfKttLearuWrP9MDH5MBPbIqV92AaeXatLxBI9gBaebbnrfifHhDYfgasaacH8akY=wiFfYdH8Gipec8Eeeu0xXdbba9frFj0=OqFfea0dXdd9vqai=hGuQ8kuc9pgc9s8qqaq=dirpe0xb9q8qiLsFr0=vr0=vr0dc8meaabaqaciaacaGaaeqabaqabeGadaaakeaacuWGTbqBgaqeaaaa@2E27@

*g *refers to the number of clusters within each arm and m¯
 MathType@MTEF@5@5@+=feaafiart1ev1aaatCvAUfKttLearuWrP9MDH5MBPbIqV92AaeXatLxBI9gBaebbnrfifHhDYfgasaacH8akY=wiFfYdH8Gipec8Eeeu0xXdbba9frFj0=OqFfea0dXdd9vqai=hGuQ8kuc9pgc9s8qqaq=dirpe0xb9q8qiLsFr0=vr0=vr0dc8meaabaqaciaacaGaaeqabaqabeGadaaakeaacuWGTbqBgaqeaaaa@2E27@ is the average cluster size

*Equal weights *(*denoted w*_1_) [3]

With an equal weights correction, the VIF is expressed as:

VIFw1=m¯g∑j=1g1mj(1−ρ)+m¯ρ
 MathType@MTEF@5@5@+=feaafiart1ev1aaatCvAUfKttLearuWrP9MDH5MBPbIqV92AaeXatLxBI9gBaebbnrfifHhDYfgasaacH8akY=wiFfYdH8Gipec8Eeeu0xXdbba9frFj0=OqFfea0dXdd9vqai=hGuQ8kuc9pgc9s8qqaq=dirpe0xb9q8qiLsFr0=vr0=vr0dc8meaabaqaciaacaGaaeqabaqabeGadaaakeaacqWGwbGvcqWGjbqscqWGgbGrdaWgaaWcbaGaem4DaC3aaSbaaWqaaiabigdaXaqabaaaleqaaOGaeyypa0ZaaSaaaeaacuWGTbqBgaqeaaqaaiabdEgaNbaadaaeWbqaamaalaaabaGaeGymaedabaGaemyBa02aaSbaaSqaaiabdQgaQbqabaaaaOWaaeWaaeaacqaIXaqmcqGHsisliiGacqWFbpGCaiaawIcacaGLPaaacqGHRaWkcuWGTbqBgaqeaiab=f8aYbWcbaGaemOAaOMaeyypa0JaeGymaedabaGaem4zaCganiabggHiLdaaaa@4AF5@ where m¯=1g∑j=1gmj
 MathType@MTEF@5@5@+=feaafiart1ev1aaatCvAUfKttLearuWrP9MDH5MBPbIqV92AaeXatLxBI9gBaebbnrfifHhDYfgasaacH8akY=wiFfYdH8Gipec8Eeeu0xXdbba9frFj0=OqFfea0dXdd9vqai=hGuQ8kuc9pgc9s8qqaq=dirpe0xb9q8qiLsFr0=vr0=vr0dc8meaabaqaciaacaGaaeqabaqabeGadaaakeaacuWGTbqBgaqeaiabg2da9maalaaabaGaeGymaedabaGaem4zaCgaamaaqahabaGaemyBa02aaSbaaSqaaiabdQgaQbqabaaabaGaemOAaOMaeyypa0JaeGymaedabaGaem4zaCganiabggHiLdaaaa@3B51@

With a Pareto imbalance, this equation is expressed as:

VIFw1=m¯w1g(0.8g0.25m¯w1+0.2g4m¯w1)(1−ρ)+m¯w1ρ=3.25+(m¯w1−3.25)ρ
 MathType@MTEF@5@5@+=feaafiart1ev1aaatCvAUfKttLearuWrP9MDH5MBPbIqV92AaeXatLxBI9gBaebbnrfifHhDYfgasaacH8akY=wiFfYdH8Gipec8Eeeu0xXdbba9frFj0=OqFfea0dXdd9vqai=hGuQ8kuc9pgc9s8qqaq=dirpe0xb9q8qiLsFr0=vr0=vr0dc8meaabaqaciaacaGaaeqabaqabeGadaaakqaaeeqaaiabdAfawjabdMeajjabdAeagnaaBaaaleaacqWG3bWDdaWgaaadbaGaeGymaedabeaaaSqabaGccqGH9aqpdaWcaaqaaiqbd2gaTzaaraWaaSbaaSqaaiabdEha3naaBaaameaacqaIXaqmaeqaaaWcbeaaaOqaaiabdEgaNbaadaqadaqaamaalaaabaGaeGimaaJaeiOla4IaeGioaGJaem4zaCgabaGaeGimaaJaeiOla4IaeGOmaiJaeGynauJafmyBa0MbaebadaWgaaWcbaGaem4DaC3aaSbaaWqaaiabigdaXaqabaaaleqaaaaakiabgUcaRmaalaaabaGaeGimaaJaeiOla4IaeGOmaiJaem4zaCgabaGaeGinaqJafmyBa0MbaebadaWgaaWcbaGaem4DaC3aaSbaaWqaaiabigdaXaqabaaaleqaaaaaaOGaayjkaiaawMcaamaabmaabaGaeGymaeJaeyOeI0ccciGae8xWdihacaGLOaGaayzkaaGaey4kaSIafmyBa0MbaebadaWgaaWcbaGaem4DaC3aaSbaaWqaaiabigdaXaqabaaaleqaaOGae8xWdihabaaccaGae4xpa0Jae43mamJae4Nla4Iae4NmaiJae4xnauJae43kaSYaaeWaaeaacuWGTbqBgaqeamaaBaaaleaacqWG3bWDdaWgaaadbaGaeGymaedabeaaaSqabaGccqGHsislcqaIZaWmcqGGUaGlcqaIYaGmcqaI1aqnaiaawIcacaGLPaaacqWFbpGCaaaa@6FAB@

where m¯w1
 MathType@MTEF@5@5@+=feaafiart1ev1aaatCvAUfKttLearuWrP9MDH5MBPbIqV92AaeXatLxBI9gBaebbnrfifHhDYfgasaacH8akY=wiFfYdH8Gipec8Eeeu0xXdbba9frFj0=OqFfea0dXdd9vqai=hGuQ8kuc9pgc9s8qqaq=dirpe0xb9q8qiLsFr0=vr0=vr0dc8meaabaqaciaacaGaaeqabaqabeGadaaakeaacuWGTbqBgaqeamaaBaaaleaacqWG3bWDdaWgaaadbaGaeGymaedabeaaaSqabaaaaa@30F2@ (the average cluster size for which an equal weights correction is used) is defined as:

m¯w1g=2T2ES2[3.25+(m¯w1−3.25)ρ]
 MathType@MTEF@5@5@+=feaafiart1ev1aaatCvAUfKttLearuWrP9MDH5MBPbIqV92AaeXatLxBI9gBaebbnrfifHhDYfgasaacH8akY=wiFfYdH8Gipec8Eeeu0xXdbba9frFj0=OqFfea0dXdd9vqai=hGuQ8kuc9pgc9s8qqaq=dirpe0xb9q8qiLsFr0=vr0=vr0dc8meaabaqaciaacaGaaeqabaqabeGadaaakeaacuWGTbqBgaqeamaaBaaaleaacqWG3bWDdaWgaaadbaGaeGymaedabeaaaSqabaGccqWGNbWzcqGH9aqpdaWcaaqaaiabikdaYiabdsfaunaaCaaaleqabaGaeGOmaidaaaGcbaGaemyrauKaem4uam1aaWbaaSqabeaacqaIYaGmaaaaaOWaamWaaeaacqaIZaWmcqGGUaGlcqaIYaGmcqaI1aqncqGHRaWkdaqadaqaaiqbd2gaTzaaraWaaSbaaSqaaiabdEha3naaBaaameaacqaIXaqmaeqaaaWcbeaakiabgkHiTiabiodaZiabc6caUiabikdaYiabiwda1aGaayjkaiaawMcaaGGaciab=f8aYbGaay5waiaaw2faaaaa@4D05@

which leads to :

m¯w1=6.5(1−ρ)T2gES2−2ρT2,
 MathType@MTEF@5@5@+=feaafiart1ev1aaatCvAUfKttLearuWrP9MDH5MBPbIqV92AaeXatLxBI9gBaebbnrfifHhDYfgasaacH8akY=wiFfYdH8Gipec8Eeeu0xXdbba9frFj0=OqFfea0dXdd9vqai=hGuQ8kuc9pgc9s8qqaq=dirpe0xb9q8qiLsFr0=vr0=vr0dc8meaabaqaciaacaGaaeqabaqabeGadaaakeaacuWGTbqBgaqeamaaBaaaleaacqWG3bWDdaWgaaadbaGaeGymaedabeaaaSqabaGccqGH9aqpdaWcaaqaaiabiAda2iabc6caUiabiwda1maabmaabaGaeGymaeJaeyOeI0ccciGae8xWdihacaGLOaGaayzkaaGaemivaq1aaWbaaSqabeaacqaIYaGmaaaakeaacqWGNbWzcqWGfbqrcqWGtbWudaahaaWcbeqaaiabikdaYaaakiabgkHiTiabikdaYiab=f8aYjabdsfaunaaCaaaleqabaGaeGOmaidaaaaakiabcYcaSaaa@4805@

where *ES *refers to the effect size and *T *= *t*_(1 - *α*/2),2(*g *- 1) _+ *t*_(1 - *β*),2(*g *- 1)_

*Cluster size weights *(*denoted w*_2_) [3]

VIFw2=1+(mA¯−1)ρ
 MathType@MTEF@5@5@+=feaafiart1ev1aaatCvAUfKttLearuWrP9MDH5MBPbIqV92AaeXatLxBI9gBaebbnrfifHhDYfgasaacH8akY=wiFfYdH8Gipec8Eeeu0xXdbba9frFj0=OqFfea0dXdd9vqai=hGuQ8kuc9pgc9s8qqaq=dirpe0xb9q8qiLsFr0=vr0=vr0dc8meaabaqaciaacaGaaeqabaqabeGadaaakeaacqWGwbGvcqWGjbqscqWGgbGrdaWgaaWcbaGaem4DaC3aaSbaaWqaaiabikdaYaqabaaaleqaaOGaeyypa0JaeGymaeJaey4kaSIaeiikaGYaa0aaaeaacqWGTbqBdaWgaaWcbaGaemyqaeeabeaaaaGccqGHsislcqaIXaqmcqGGPaqkiiGacqWFbpGCaaa@3DCB@ where mA¯=∑j=1gmj2∑j=1gmj
 MathType@MTEF@5@5@+=feaafiart1ev1aaatCvAUfKttLearuWrP9MDH5MBPbIqV92AaeXatLxBI9gBaebbnrfifHhDYfgasaacH8akY=wiFfYdH8Gipec8Eeeu0xXdbba9frFj0=OqFfea0dXdd9vqai=hGuQ8kuc9pgc9s8qqaq=dirpe0xb9q8qiLsFr0=vr0=vr0dc8meaabaqaciaacaGaaeqabaqabeGadaaakeaadaqdaaqaaiabd2gaTnaaBaaaleaacqWGbbqqaeqaaaaakiabg2da9maalaaabaWaaabCaeaacqWGTbqBdaqhaaWcbaGaemOAaOgabaGaeGOmaidaaaqaaiabdQgaQjabg2da9iabigdaXaqaaiabdEgaNbqdcqGHris5aaGcbaWaaabCaeaacqWGTbqBdaWgaaWcbaGaemOAaOgabeaaaeaacqWGQbGAcqGH9aqpcqaIXaqmaeaacqWGNbWza0GaeyyeIuoaaaaaaa@450E@

With a Pareto imbalance, we can write the equation as:

m¯A=0.8g(0.25m¯w2)2+0.2g(4m¯w2)2m¯w2g=3.25m¯w2
 MathType@MTEF@5@5@+=feaafiart1ev1aaatCvAUfKttLearuWrP9MDH5MBPbIqV92AaeXatLxBI9gBaebbnrfifHhDYfgasaacH8akY=wiFfYdH8Gipec8Eeeu0xXdbba9frFj0=OqFfea0dXdd9vqai=hGuQ8kuc9pgc9s8qqaq=dirpe0xb9q8qiLsFr0=vr0=vr0dc8meaabaqaciaacaGaaeqabaqabeGadaaakeaacuWGTbqBgaqeamaaBaaaleaacqWGbbqqaeqaaOGaeyypa0ZaaSaaaeaacqaIWaamcqGGUaGlcqaI4aaocqWGNbWzdaqadaqaaiabicdaWiabc6caUiabikdaYiabiwda1iqbd2gaTzaaraWaaSbaaSqaaiabdEha3naaBaaameaacqaIYaGmaeqaaaWcbeaaaOGaayjkaiaawMcaamaaCaaaleqabaGaeGOmaidaaOGaey4kaSIaeGimaaJaeiOla4IaeGOmaiJaem4zaC2aaeWaaeaacqaI0aancuWGTbqBgaqeamaaBaaaleaacqWG3bWDdaWgaaadbaGaeGOmaidabeaaaSqabaaakiaawIcacaGLPaaadaahaaWcbeqaaiabikdaYaaaaOqaaiqbd2gaTzaaraWaaSbaaSqaaiabdEha3naaBaaameaacqaIYaGmaeqaaaWcbeaakiabdEgaNbaacqGH9aqpcqaIZaWmcqGGUaGlcqaIYaGmcqaI1aqncuWGTbqBgaqeamaaBaaaleaacqWG3bWDdaWgaaadbaGaeGOmaidabeaaaSqabaaaaa@5B15@

So the VIF is reduced to:

VIFw2=1+(3.25m¯w2−1)ρ     (6)
 MathType@MTEF@5@5@+=feaafiart1ev1aaatCvAUfKttLearuWrP9MDH5MBPbIqV92AaeXatLxBI9gBaebbnrfifHhDYfgasaacH8akY=wiFfYdH8Gipec8Eeeu0xXdbba9frFj0=OqFfea0dXdd9vqai=hGuQ8kuc9pgc9s8qqaq=dirpe0xb9q8qiLsFr0=vr0=vr0dc8meaabaqaciaacaGaaeqabaqabeGadaaakeaacqWGwbGvcqWGjbqscqWGgbGrdaWgaaWcbaGaem4DaC3aaSbaaWqaaiabikdaYaqabaaaleqaaOGaeyypa0JaeGymaeJaey4kaSYaaeWaaeaacqaIZaWmcqGGUaGlcqaIYaGmcqaI1aqncuWGTbqBgaqeamaaBaaaleaacqWG3bWDdaWgaaadbaGaeGOmaidabeaaaSqabaGccqGHsislcqaIXaqmaiaawIcacaGLPaaaiiGacqWFbpGCcaWLjaGaaCzcamaabmaabaGaeGOnaydacaGLOaGaayzkaaaaaa@46C8@

and

m¯w2=2(1−ρ)T2gES2−6.5ρT2
 MathType@MTEF@5@5@+=feaafiart1ev1aaatCvAUfKttLearuWrP9MDH5MBPbIqV92AaeXatLxBI9gBaebbnrfifHhDYfgasaacH8akY=wiFfYdH8Gipec8Eeeu0xXdbba9frFj0=OqFfea0dXdd9vqai=hGuQ8kuc9pgc9s8qqaq=dirpe0xb9q8qiLsFr0=vr0=vr0dc8meaabaqaciaacaGaaeqabaqabeGadaaakeaacuWGTbqBgaqeamaaBaaaleaacqWG3bWDdaWgaaadbaGaeGOmaidabeaaaSqabaGccqGH9aqpdaWcaaqaaiabikdaYmaabmaabaGaeGymaeJaeyOeI0ccciGae8xWdihacaGLOaGaayzkaaGaemivaq1aaWbaaSqabeaacqaIYaGmaaaakeaacqWGNbWzcqWGfbqrcqWGtbWudaahaaWcbeqaaiabikdaYaaakiabgkHiTiabiAda2iabc6caUiabiwda1iab=f8aYjabdsfaunaaCaaaleqabaGaeGOmaidaaaaaaaa@471D@

*Minimum variance weights *(*denoted w*_3_) [3]

VIFw3=m¯g∑j=1gmj1+(mj−1)ρ
 MathType@MTEF@5@5@+=feaafiart1ev1aaatCvAUfKttLearuWrP9MDH5MBPbIqV92AaeXatLxBI9gBaebbnrfifHhDYfgasaacH8akY=wiFfYdH8Gipec8Eeeu0xXdbba9frFj0=OqFfea0dXdd9vqai=hGuQ8kuc9pgc9s8qqaq=dirpe0xb9q8qiLsFr0=vr0=vr0dc8meaabaqaciaacaGaaeqabaqabeGadaaakeaacqWGwbGvcqWGjbqscqWGgbGrdaWgaaWcbaGaem4DaC3aaSbaaWqaaiabiodaZaqabaaaleqaaOGaeyypa0ZaaSaaaeaacuWGTbqBgaqeaiabdEgaNbqaamaaqahabaWaaSaaaeaacqWGTbqBdaWgaaWcbaGaemOAaOgabeaaaOqaaiabigdaXiabgUcaRmaabmaabaGaemyBa02aaSbaaSqaaiabdQgaQbqabaGccqGHsislcqaIXaqmaiaawIcacaGLPaaaiiGacqWFbpGCaaaaleaacqWGQbGAcqGH9aqpcqaIXaqmaeaacqWGNbWza0GaeyyeIuoaaaaaaa@4AB9@

With a Pareto imbalance, the equation can be written as:

VIFw3=m¯w3g0.8g0.25m¯w31+(0.25m¯w3−1)ρ+0.2g4m¯w31+(4m¯w3−1)ρ=(1+(4m¯w3−1)ρ)(1+(0.25m¯w3−1)ρ)1+(m¯w3−1)ρ
 MathType@MTEF@5@5@+=feaafiart1ev1aaatCvAUfKttLearuWrP9MDH5MBPbIqV92AaeXatLxBI9gBaebbnrfifHhDYfgasaacH8akY=wiFfYdH8Gipec8Eeeu0xXdbba9frFj0=OqFfea0dXdd9vqai=hGuQ8kuc9pgc9s8qqaq=dirpe0xb9q8qiLsFr0=vr0=vr0dc8meaabaqaciaacaGaaeqabaqabeGadaaakqaaeeqaaiabdAfawjabdMeajjabdAeagnaaBaaaleaacqWG3bWDdaWgaaadbaGaeG4mamdabeaaaSqabaGccqGH9aqpdaWcaaqaaiqbd2gaTzaaraWaaSbaaSqaaiabdEha3naaBaaameaacqaIZaWmaeqaaaWcbeaakiabdEgaNbqaaiabicdaWiabc6caUiabiIda4iabdEgaNnaalaaabaGaeGimaaJaeiOla4IaeGOmaiJaeGynauJafmyBa0MbaebadaWgaaWcbaGaem4DaC3aaSbaaWqaaiabiodaZaqabaaaleqaaaGcbaGaeGymaeJaey4kaSYaaeWaaeaacqaIWaamcqGGUaGlcqaIYaGmcqaI1aqncuWGTbqBgaqeamaaBaaaleaacqWG3bWDdaWgaaadbaGaeG4mamdabeaaaSqabaGccqGHsislcqaIXaqmaiaawIcacaGLPaaaiiGacqWFbpGCaaGaey4kaSIaeGimaaJaeiOla4IaeGOmaiJaem4zaC2aaSaaaeaacqaI0aancuWGTbqBgaqeamaaBaaaleaacqWG3bWDdaWgaaadbaGaeG4mamdabeaaaSqabaaakeaacqaIXaqmcqGHRaWkdaqadaqaaiabisda0iqbd2gaTzaaraWaaSbaaSqaaiabdEha3naaBaaameaacqaIZaWmaeqaaaWcbeaakiabgkHiTiabigdaXaGaayjkaiaawMcaaiab=f8aYbaaaaaabaGaeyypa0ZaaSaaaeaadaqadaqaaiabigdaXiabgUcaRmaabmaabaGaeGinaqJafmyBa0MbaebadaWgaaWcbaGaem4DaC3aaSbaaWqaaiabiodaZaqabaaaleqaaOGaeyOeI0IaeGymaedacaGLOaGaayzkaaGae8xWdihacaGLOaGaayzkaaWaaeWaaeaacqaIXaqmcqGHRaWkdaqadaqaaiabicdaWiabc6caUiabikdaYiabiwda1iqbd2gaTzaaraWaaSbaaSqaaiabdEha3naaBaaameaacqaIZaWmaeqaaaWcbeaakiabgkHiTiabigdaXaGaayjkaiaawMcaaiab=f8aYbGaayjkaiaawMcaaaqaaiabigdaXiabgUcaRmaabmaabaGafmyBa0MbaebadaWgaaWcbaGaem4DaC3aaSbaaWqaaiabiodaZaqabaaaleqaaOGaeyOeI0IaeGymaedacaGLOaGaayzkaaGae8xWdihaaaaaaa@964E@

with

m¯w3g=2T2ES2[(1+(4m¯w3−1)ρ)(1+(0.25m¯w3−1)ρ)1+(m¯w3−1)ρ]
 MathType@MTEF@5@5@+=feaafiart1ev1aaatCvAUfKttLearuWrP9MDH5MBPbIqV92AaeXatLxBI9gBaebbnrfifHhDYfgasaacH8akY=wiFfYdH8Gipec8Eeeu0xXdbba9frFj0=OqFfea0dXdd9vqai=hGuQ8kuc9pgc9s8qqaq=dirpe0xb9q8qiLsFr0=vr0=vr0dc8meaabaqaciaacaGaaeqabaqabeGadaaakeaacuWGTbqBgaqeamaaBaaaleaacqWG3bWDdaWgaaadbaGaeG4mamdabeaaaSqabaGccqWGNbWzcqGH9aqpdaWcaaqaaiabikdaYiabdsfaunaaCaaaleqabaGaeGOmaidaaaGcbaGaemyrauKaem4uam1aaWbaaSqabeaacqaIYaGmaaaaaOWaamWaaeaadaWcaaqaamaabmaabaGaeGymaeJaey4kaSYaaeWaaeaacqaI0aancuWGTbqBgaqeamaaBaaaleaacqWG3bWDdaWgaaadbaGaeG4mamdabeaaaSqabaGccqGHsislcqaIXaqmaiaawIcacaGLPaaaiiGacqWFbpGCaiaawIcacaGLPaaadaqadaqaaiabigdaXiabgUcaRmaabmaabaGaeGimaaJaeiOla4IaeGOmaiJaeGynauJafmyBa0MbaebadaWgaaWcbaGaem4DaC3aaSbaaWqaaiabiodaZaqabaaaleqaaOGaeyOeI0IaeGymaedacaGLOaGaayzkaaGae8xWdihacaGLOaGaayzkaaaabaGaeGymaeJaey4kaSYaaeWaaeaacuWGTbqBgaqeamaaBaaaleaacqWG3bWDdaWgaaadbaGaeG4mamdabeaaaSqabaGccqGHsislcqaIXaqmaiaawIcacaGLPaaacqWFbpGCaaaacaGLBbGaayzxaaaaaa@65CB@

which leads to m¯w3
 MathType@MTEF@5@5@+=feaafiart1ev1aaatCvAUfKttLearuWrP9MDH5MBPbIqV92AaeXatLxBI9gBaebbnrfifHhDYfgasaacH8akY=wiFfYdH8Gipec8Eeeu0xXdbba9frFj0=OqFfea0dXdd9vqai=hGuQ8kuc9pgc9s8qqaq=dirpe0xb9q8qiLsFr0=vr0=vr0dc8meaabaqaciaacaGaaeqabaqabeGadaaakeaacuWGTbqBgaqeamaaBaaaleaacqWG3bWDdaWgaaadbaGaeG4mamdabeaaaSqabaaaaa@30F6@ being the positive solution of the following equation:

m¯w32ρ[gES2−2ρT2]+m¯w3(1−ρ)[gES2−8.5ρT2]−2(1−ρ)2T2=0
 MathType@MTEF@5@5@+=feaafiart1ev1aaatCvAUfKttLearuWrP9MDH5MBPbIqV92AaeXatLxBI9gBaebbnrfifHhDYfgasaacH8akY=wiFfYdH8Gipec8Eeeu0xXdbba9frFj0=OqFfea0dXdd9vqai=hGuQ8kuc9pgc9s8qqaq=dirpe0xb9q8qiLsFr0=vr0=vr0dc8meaabaqaciaacaGaaeqabaqabeGadaaakeaacuWGTbqBgaqeamaaDaaaleaacqWG3bWDdaWgaaadbaGaeG4mamdabeaaaSqaaiabikdaYaaaiiGakiab=f8aYnaadmaabaGaem4zaCMaemyrauKaem4uam1aaWbaaSqabeaacqaIYaGmaaGccqGHsislcqaIYaGmcqWFbpGCcqWGubavdaahaaWcbeqaaiabikdaYaaaaOGaay5waiaaw2faaiabgUcaRiqbd2gaTzaaraWaaSbaaSqaaiabdEha3naaBaaameaacqaIZaWmaeqaaaWcbeaakmaabmaabaGaeGymaeJaeyOeI0Iae8xWdihacaGLOaGaayzkaaWaamWaaeaacqWGNbWzcqWGfbqrcqWGtbWudaahaaWcbeqaaiabikdaYaaakiabgkHiTiabiIda4iabc6caUiabiwda1iab=f8aYjabdsfaunaaCaaaleqabaGaeGOmaidaaaGccaGLBbGaayzxaaGaeyOeI0IaeGOmaiZaaeWaaeaacqaIXaqmcqGHsislcqWFbpGCaiaawIcacaGLPaaadaahaaWcbeqaaiabikdaYaaakiabdsfaunaaCaaaleqabaGaeGOmaidaaOGaeyypa0JaeGimaadaaa@65C0@

*Distribution-based correction *(*denoted d*) [4]

VIFd=1+(E(m)2+var⁡(m)E(m)−1)ρ
 MathType@MTEF@5@5@+=feaafiart1ev1aaatCvAUfKttLearuWrP9MDH5MBPbIqV92AaeXatLxBI9gBaebbnrfifHhDYfgasaacH8akY=wiFfYdH8Gipec8Eeeu0xXdbba9frFj0=OqFfea0dXdd9vqai=hGuQ8kuc9pgc9s8qqaq=dirpe0xb9q8qiLsFr0=vr0=vr0dc8meaabaqaciaacaGaaeqabaqabeGadaaakeaacqWGwbGvcqWGjbqscqWGgbGrdaWgaaWcbaGaemizaqgabeaakiabg2da9iabigdaXiabgUcaRmaabmaabaWaaSaaaeaacqWGfbqrdaqadaqaaiabd2gaTbGaayjkaiaawMcaamaaCaaaleqabaGaeGOmaidaaOGaey4kaSIagiODayNaeiyyaeMaeiOCai3aaeWaaeaacqWGTbqBaiaawIcacaGLPaaaaeaacqWGfbqrdaqadaqaaiabd2gaTbGaayjkaiaawMcaaaaacqGHsislcqaIXaqmaiaawIcacaGLPaaaiiGacqWFbpGCaaa@4ACE@



So we have:



with

m¯dg=2T2ES2[1+(3.25m¯d−1)ρ]
 MathType@MTEF@5@5@+=feaafiart1ev1aaatCvAUfKttLearuWrP9MDH5MBPbIqV92AaeXatLxBI9gBaebbnrfifHhDYfgasaacH8akY=wiFfYdH8Gipec8Eeeu0xXdbba9frFj0=OqFfea0dXdd9vqai=hGuQ8kuc9pgc9s8qqaq=dirpe0xb9q8qiLsFr0=vr0=vr0dc8meaabaqaciaacaGaaeqabaqabeGadaaakeaacuWGTbqBgaqeamaaBaaaleaacqWGKbazaeqaaOGaem4zaCMaeyypa0ZaaSaaaeaacqaIYaGmcqWGubavdaahaaWcbeqaaiabikdaYaaaaOqaaiabdweafjabdofatnaaCaaaleqabaGaeGOmaidaaaaakmaadmaabaGaeGymaeJaey4kaSYaaeWaaeaacqaIZaWmcqGGUaGlcqaIYaGmcqaI1aqncuWGTbqBgaqeamaaBaaaleaacqWGKbazaeqaaOGaeyOeI0IaeGymaedacaGLOaGaayzkaaacciGae8xWdihacaGLBbGaayzxaaaaaa@4887@

that is to say:

m¯d=2(1−ρ)T2gES2−6.5ρT2
 MathType@MTEF@5@5@+=feaafiart1ev1aaatCvAUfKttLearuWrP9MDH5MBPbIqV92AaeXatLxBI9gBaebbnrfifHhDYfgasaacH8akY=wiFfYdH8Gipec8Eeeu0xXdbba9frFj0=OqFfea0dXdd9vqai=hGuQ8kuc9pgc9s8qqaq=dirpe0xb9q8qiLsFr0=vr0=vr0dc8meaabaqaciaacaGaaeqabaqabeGadaaakeaacuWGTbqBgaqeamaaBaaaleaacqWGKbazaeqaaOGaeyypa0ZaaSaaaeaacqaIYaGmdaqadaqaaiabigdaXiabgkHiTGGaciab=f8aYbGaayjkaiaawMcaaiabdsfaunaaCaaaleqabaGaeGOmaidaaaGcbaGaem4zaCMaemyrauKaem4uam1aaWbaaSqabeaacqaIYaGmaaGccqGHsislcqaI2aGncqGGUaGlcqaI1aqncqWFbpGCcqWGubavdaahaaWcbeqaaiabikdaYaaaaaaaaa@45CD@

One then recognizes the results obtained using the cluster size weights correction.

## Appendix B: minimum variance weights-corrected variance inflation factor (VIF) for an *a priori *postulated Pareto-like imbalance

Characteristics of the Pareto-like imbalance

**Table 6:** 

	Number of clusters by intervention arm	Number of patients belonging to the clusters	Mean cluster size
Small clusters	(1-*γ*)*g*	(1 - *τ*)m¯ MathType@MTEF@5@5@+=feaafiart1ev1aaatCvAUfKttLearuWrP9MDH5MBPbIqV92AaeXatLxBI9gBaebbnrfifHhDYfgasaacH8akY=wiFfYdH8Gipec8Eeeu0xXdbba9frFj0=OqFfea0dXdd9vqai=hGuQ8kuc9pgc9s8qqaq=dirpe0xb9q8qiLsFr0=vr0=vr0dc8meaabaqaciaacaGaaeqabaqabeGadaaakeaacuWGTbqBgaqeaaaa@2E27@*g*	1−τ1−γm¯ MathType@MTEF@5@5@+=feaafiart1ev1aaatCvAUfKttLearuWrP9MDH5MBPbIqV92AaeXatLxBI9gBaebbnrfifHhDYfgasaacH8akY=wiFfYdH8Gipec8Eeeu0xXdbba9frFj0=OqFfea0dXdd9vqai=hGuQ8kuc9pgc9s8qqaq=dirpe0xb9q8qiLsFr0=vr0=vr0dc8meaabaqaciaacaGaaeqabaqabeGadaaakeaadaWcaaqaaiabigdaXiabgkHiTGGaciab=r8a0bqaaiabigdaXiabgkHiTiab=n7aNbaacuWGTbqBgaqeaaaa@355F@
Big clusters	*γg*	*τ*m¯ MathType@MTEF@5@5@+=feaafiart1ev1aaatCvAUfKttLearuWrP9MDH5MBPbIqV92AaeXatLxBI9gBaebbnrfifHhDYfgasaacH8akY=wiFfYdH8Gipec8Eeeu0xXdbba9frFj0=OqFfea0dXdd9vqai=hGuQ8kuc9pgc9s8qqaq=dirpe0xb9q8qiLsFr0=vr0=vr0dc8meaabaqaciaacaGaaeqabaqabeGadaaakeaacuWGTbqBgaqeaaaa@2E27@*g*	τγm¯ MathType@MTEF@5@5@+=feaafiart1ev1aaatCvAUfKttLearuWrP9MDH5MBPbIqV92AaeXatLxBI9gBaebbnrfifHhDYfgasaacH8akY=wiFfYdH8Gipec8Eeeu0xXdbba9frFj0=OqFfea0dXdd9vqai=hGuQ8kuc9pgc9s8qqaq=dirpe0xb9q8qiLsFr0=vr0=vr0dc8meaabaqaciaacaGaaeqabaqabeGadaaakeaadaWcaaqaaGGaciab=r8a0bqaaiab=n7aNbaacuWGTbqBgaqeaaaa@31A5@

*g *refers to the number of clusters within each arm and m¯
 MathType@MTEF@5@5@+=feaafiart1ev1aaatCvAUfKttLearuWrP9MDH5MBPbIqV92AaeXatLxBI9gBaebbnrfifHhDYfgasaacH8akY=wiFfYdH8Gipec8Eeeu0xXdbba9frFj0=OqFfea0dXdd9vqai=hGuQ8kuc9pgc9s8qqaq=dirpe0xb9q8qiLsFr0=vr0=vr0dc8meaabaqaciaacaGaaeqabaqabeGadaaakeaacuWGTbqBgaqeaaaa@2E27@ is the average cluster size

Minimum variance weighs VIF

VIFw3=m¯g∑j=1gmj1+(mj−1)ρ=m¯g(1−γ)g1−τ1−γm¯1+(1−τ1−γm¯−1)ρ+γgτγm¯1+(τγm¯−1)ρ
 MathType@MTEF@5@5@+=feaafiart1ev1aaatCvAUfKttLearuWrP9MDH5MBPbIqV92AaeXatLxBI9gBaebbnrfifHhDYfgasaacH8akY=wiFfYdH8Gipec8Eeeu0xXdbba9frFj0=OqFfea0dXdd9vqai=hGuQ8kuc9pgc9s8qqaq=dirpe0xb9q8qiLsFr0=vr0=vr0dc8meaabaqaciaacaGaaeqabaqabeGadaaakqaaeeqaaiabdAfawjabdMeajjabdAeagnaaBaaaleaacqWG3bWDdaWgaaadbaGaeG4mamdabeaaaSqabaGccqGH9aqpdaWcaaqaaiqbd2gaTzaaraGaem4zaCgabaWaaabCaeaadaWcaaqaaiabd2gaTnaaBaaaleaacqWGQbGAaeqaaaGcbaGaeGymaeJaey4kaSYaaeWaaeaacqWGTbqBdaWgaaWcbaGaemOAaOgabeaakiabgkHiTiabigdaXaGaayjkaiaawMcaaGGaciab=f8aYbaaaSqaaiabdQgaQjabg2da9iabigdaXaqaaiabdEgaNbqdcqGHris5aaaaaOqaaiabg2da9maalaaabaGafmyBa0MbaebacqWGNbWzaeaadaqadaqaaiabigdaXiabgkHiTiab=n7aNbGaayjkaiaawMcaaiabdEgaNnaalaaabaWaaSaaaeaacqaIXaqmcqGHsislcqWFepaDaeaacqaIXaqmcqGHsislcqWFZoWzaaGafmyBa0MbaebaaeaacqaIXaqmcqGHRaWkdaqadaqaamaalaaabaGaeGymaeJaeyOeI0Iae8hXdqhabaGaeGymaeJaeyOeI0Iae83SdCgaaiqbd2gaTzaaraGaeyOeI0IaeGymaedacaGLOaGaayzkaaGae8xWdihaaiabgUcaRiab=n7aNjabdEgaNnaalaaabaWaaSaaaeaacqWFepaDaeaacqWFZoWzaaGafmyBa0MbaebaaeaacqaIXaqmcqGHRaWkdaqadaqaamaalaaabaGae8hXdqhabaGae83SdCgaaiqbd2gaTzaaraGaeyOeI0IaeGymaedacaGLOaGaayzkaaGae8xWdihaaaaaaaaa@821D@

So we obtain:

VIF=[1+(1−τ1−γm−1)ρ][1+(τγm−1)ρ]τ[1+(1−τ1−γm−1)ρ]+(1−τ)[1+(τγm−1)ρ]
 MathType@MTEF@5@5@+=feaafiart1ev1aaatCvAUfKttLearuWrP9MDH5MBPbIqV92AaeXatLxBI9gBaebbnrfifHhDYfgasaacH8akY=wiFfYdH8Gipec8Eeeu0xXdbba9frFj0=OqFfea0dXdd9vqai=hGuQ8kuc9pgc9s8qqaq=dirpe0xb9q8qiLsFr0=vr0=vr0dc8meaabaqaciaacaGaaeqabaqabeGadaaakeaacqWGwbGvcqWGjbqscqWGgbGrcqGH9aqpdaWcaaqaamaadmaabaGaeGymaeJaey4kaSYaaeWaaeaadaWcaaqaaiabigdaXiabgkHiTGGaciab=r8a0bqaaiabigdaXiabgkHiTiab=n7aNbaacqWGTbqBcqGHsislcqaIXaqmaiaawIcacaGLPaaacqWFbpGCaiaawUfacaGLDbaadaWadaqaaiabigdaXiabgUcaRmaabmaabaWaaSaaaeaacqWFepaDaeaacqWFZoWzaaGaemyBa0MaeyOeI0IaeGymaedacaGLOaGaayzkaaGae8xWdihacaGLBbGaayzxaaaabaGae8hXdq3aamWaaeaacqaIXaqmcqGHRaWkdaqadaqaamaalaaabaGaeGymaeJaeyOeI0Iae8hXdqhabaGaeGymaeJaeyOeI0Iae83SdCgaaiabd2gaTjabgkHiTiabigdaXaGaayjkaiaawMcaaiab=f8aYbGaay5waiaaw2faaiabgUcaRmaabmaabaGaeGymaeJaeyOeI0Iae8hXdqhacaGLOaGaayzkaaWaamWaaeaacqaIXaqmcqGHRaWkdaqadaqaamaalaaabaGae8hXdqhabaGae83SdCgaaiabd2gaTjabgkHiTiabigdaXaGaayjkaiaawMcaaiab=f8aYbGaay5waiaaw2faaaaaaaa@7757@

## Appendix C: gini coefficient for an *a priori *postulated Pareto-like imbalance

gini=12g2m¯∑i=1g∑j=1g|mi−mj|
 MathType@MTEF@5@5@+=feaafiart1ev1aaatCvAUfKttLearuWrP9MDH5MBPbIqV92AaeXatLxBI9gBaebbnrfifHhDYfgasaacH8akY=wiFfYdH8Gipec8Eeeu0xXdbba9frFj0=OqFfea0dXdd9vqai=hGuQ8kuc9pgc9s8qqaq=dirpe0xb9q8qiLsFr0=vr0=vr0dc8meaabaqaciaacaGaaeqabaqabeGadaaakeaacqWGNbWzcqWGPbqAcqWGUbGBcqWGPbqAcqGH9aqpdaWcaaqaaiabigdaXaqaaiabikdaYiabdEgaNnaaCaaaleqabaGaeGOmaidaaOGafmyBa0MbaebaaaWaaabCaeaadaaeWbqaamaaemaabaGaemyBa02aaSbaaSqaaiabdMgaPbqabaGccqGHsislcqWGTbqBdaWgaaWcbaGaemOAaOgabeaaaOGaay5bSlaawIa7aaWcbaGaemOAaOMaeyypa0JaeGymaedabaGaem4zaCganiabggHiLdaaleaacqWGPbqAcqGH9aqpcqaIXaqmaeaacqWGNbWza0GaeyyeIuoaaaa@50E0@

Given the characteristics of the Pareto-like imbalance presented in appendix B, considering that clusters are ordered hierarchically by increasing size, the matrix of the difference |*m*_*i *_- *m*_*j*_| can be written as:

M=(0(γg,γg)1(γg,(1−γ)g)|τ−γγ(1−γ)|1((1−γ)g,γg)|τ−γγ(1−γ)|0((1−γ)g,(1−γ)g))
 MathType@MTEF@5@5@+=feaafiart1ev1aaatCvAUfKttLearuWrP9MDH5MBPbIqV92AaeXatLxBI9gBaebbnrfifHhDYfgasaacH8akY=wiFfYdH8Gipec8Eeeu0xXdbba9frFj0=OqFfea0dXdd9vqai=hGuQ8kuc9pgc9s8qqaq=dirpe0xb9q8qiLsFr0=vr0=vr0dc8meaabaqaciaacaGaaeqabaqabeGadaaakeaacqWGnbqtcqGH9aqpdaqadaqaauaabeqaciaaaeaacqaIWaamdaWgaaWcbaWaaeWaaeaaiiGacqWFZoWzcqWGNbWzcqGGSaalcqWFZoWzcqWGNbWzaiaawIcacaGLPaaaaeqaaaGcbaGaeGymaeZaaSbaaSqaamaabmaabaGae83SdCMaem4zaCMaeiilaWYaaeWaaeaacqaIXaqmcqGHsislcqWFZoWzaiaawIcacaGLPaaacqWGNbWzaiaawIcacaGLPaaaaeqaaOWaaqWaaeaadaWcaaqaaiab=r8a0jabgkHiTiab=n7aNbqaaiab=n7aNnaabmaabaGaeGymaeJaeyOeI0Iae83SdCgacaGLOaGaayzkaaaaaaGaay5bSlaawIa7aaqaaiabigdaXmaaBaaaleaadaqadaqaamaabmaabaGaeGymaeJaeyOeI0Iae83SdCgacaGLOaGaayzkaaGaem4zaCMaeiilaWIae83SdCMaem4zaCgacaGLOaGaayzkaaaabeaakmaaemaabaWaaSaaaeaacqWFepaDcqGHsislcqWFZoWzaeaacqWFZoWzdaqadaqaaiabigdaXiabgkHiTiab=n7aNbGaayjkaiaawMcaaaaaaiaawEa7caGLiWoaaeaacqaIWaamdaWgaaWcbaWaaeWaaeaadaqadaqaaiabigdaXiabgkHiTiab=n7aNbGaayjkaiaawMcaaiabdEgaNjabcYcaSmaabmaabaGaeGymaeJaeyOeI0Iae83SdCgacaGLOaGaayzkaaGaem4zaCgacaGLOaGaayzkaaaabeaaaaaakiaawIcacaGLPaaaaaa@8068@

Where 0_(*γg*,*γg*) _and 0_((1-*γ*)*g*,(1-*γ*)*g*) _are squared matrices of size *γg *and (1-*γ*)*g *respectively and 1_(*γg*,(1-*γ*)*g*) _and 1_((1-*γ*)*g*,*γg*) _are matices of size *γg *× (1-*γ*)*g *and (1-*γ*)*g *× *γg *respectively, containing only 1 s.

Thus:

gini=12g2m¯2g2γ(1−γ)|τ−γγ(1−γ)|m¯gini=|τ−γ|
 MathType@MTEF@5@5@+=feaafiart1ev1aaatCvAUfKttLearuWrP9MDH5MBPbIqV92AaeXatLxBI9gBaebbnrfifHhDYfgasaacH8akY=wiFfYdH8Gipec8Eeeu0xXdbba9frFj0=OqFfea0dXdd9vqai=hGuQ8kuc9pgc9s8qqaq=dirpe0xb9q8qiLsFr0=vr0=vr0dc8meaabaqaciaacaGaaeqabaqabeGadaaakqaabeqaaiabdEgaNjabdMgaPjabd6gaUjabdMgaPjabg2da9maalaaabaGaeGymaedabaGaeGOmaiJaem4zaC2aaWbaaSqabeaacqaIYaGmaaGccuWGTbqBgaqeaaaacqaIYaGmcqWGNbWzdaahaaWcbeqaaiabikdaYaaaiiGakiab=n7aNnaabmaabaGaeGymaeJaeyOeI0Iae83SdCgacaGLOaGaayzkaaWaaqWaaeaadaWcaaqaaiab=r8a0jabgkHiTiab=n7aNbqaaiab=n7aNnaabmaabaGaeGymaeJaeyOeI0Iae83SdCgacaGLOaGaayzkaaaaaaGaay5bSlaawIa7aiqbd2gaTzaaraaabaGaem4zaCMaemyAaKMaemOBa4MaemyAaKMaeyypa0ZaaqWaaeaacqWFepaDcqGHsislcqWFZoWzaiaawEa7caGLiWoaaaaa@60CF@

## Pre-publication history

The pre-publication history for this paper can be accessed here:


